# Canadian service providers' perspectives on reproductive coercion and abuse: a participatory action research to address their needs and support their actions

**DOI:** 10.1186/s12978-023-01640-w

**Published:** 2023-06-30

**Authors:** Sylvie Lévesque, Catherine Rousseau, Laurence Raynault-Rioux, Julie Laforest

**Affiliations:** 1 Sexology Department, Université du Québec à Montréal, CP 8888, Succ. Centre-Ville, Montréal, QC H3C 3P8 Canada; 2 Population Health, Interdisciplinary School of Health Sciences, University of Ottawa, Ottawa, Canada; 3 Fédération du Québec pour le Planning des Naissances, Montréal, Canada; 4 Population Health and Well-Being, Institut National de Santé Publique du Québec, Montréal, Canada

**Keywords:** Reproductive coercion and abuse, Health professionals, Intervention tools, Qualitative research, Action research

## Abstract

Reproductive coercion and abuse (RCA) is a form of violence that affects sexual and reproductive health. Women and individuals who experienced RCA in an intimate relationship frequently consult service providers (SPs), such as health professionals or violence counselors. The objective of this article, which is the result of a participative action research project targeting RCA perpetrated by in an intimate partner, is twofold: (1) to better understand the practices as well as the barriers and facilitators encountered by SPs and (2) to develop information and awareness tools with them that meet their needs. To this end, we first held focus groups with 31 SPs. The use of thematic analysis revealed intervention strategies that focus on caring and listening, identifying signs of RCA, and creating a safe environment for disclosure. Their practices also focused on harm-reduction strategies and effective referrals. Despite the importance they gave to this issue, lack of time, inappropriate settings, and inadequate training hindered them from intervening effectively with individuals who were victims of RCA. They also indicated the need for easy-to-follow practice guidelines and patient education tools. Based on these findings and the best practices identified in the grey and scientific literature, we developed a practice guide for SPs and a booklet on RCA. The development of these guide and booklets involved a lot of back and forth to meet the needs expressed by the community and health professionals.

**Supplementary Information:**

The online version contains supplementary material available at 10.1186/s12978-023-01640-w.

## Background

Reproductive coercion (RC) refers to behavior that interferes with contraception and reproductive decision-making [1]. It includes any behavior that is done to intentionally control another person's reproductive choices [2–4]. This can take the form of birth control sabotage (e.g., removing a condom without consent, destroying contraceptive pills, removing a patch or IUD) or pregnancy coercion (forcing the continuation or termination of a pregnancy) [1]. It can also involve deception by lying about infertility status, giving false information about contraception, or gaslighting women in order to interfere with their contraceptive and reproductive choices [5]. Located at the intersection of violence against women and reproductive health, these behavior acts to undermine the reproductive autonomy of the victims [6].

Up to now, reproductive coercion has been mostly studied as occurring at the hands of an intimate partner, and the term reproductive coercion and abuse (RCA) has been proposed to reflect the contexts of control, fear, and intent that are associated with this phenomenon [4]. We will be using the term RCA in this article when presenting our results. We feel it captures the essence of coercive control [7, 8] inherent to intimate partner violence (IPV).

### The importance of service providers for RCA victims

Studies in the U.S. indicate high prevalence of RCA in women [2, 9], and particularly in the 18- to 29-year age group [1]. However, RCA is not limited to the U.S., as demonstrated by a systematic international review [10]. The evidence shows that RCA perpetrated by a male partner is a serious problem worldwide that jeopardizes women’s health, integrity, and contraceptive and reproductive choices [11–14]. The impacts of RCA are multiple, beginning with the consequences for sexual and reproductive health [15, 16]. Studies have also reported consequences for emotional and psychological health, including symptoms of post-traumatic stress and emotional distress [17, 18]. Because RCA increases the risk of pregnancy and sexually transmitted diseases (STIs), many victims consult healthcare professionals for STIs screening, emergency oral contraception, pregnancy tests, or abortions [7, 19, 20]. Several will also suffer psychological consequences and consult violence counsellors or mental health specialists [21, 22]. This gives these service providers (SPs) a window of opportunity to intervene by broaching the subject of RCA as they respond to the diverse needs of women and others who come for consultations [23, 24]. SPs play a key role when women are subjected to RCA: they can provide preventive education, identify RCA, offer practical support, and refer clients to the appropriate resources.

While studies document the use of support services by individuals who have experienced RCA, few have focused on the professional practices of SPs who welcome and support these individuals. In a qualitative study examining the intervention practices of health practitioners working in a large Australian public hospital, Tarzia and colleagues [25] documented the interventions delivered in RCA situations. Health practitioners relied on their sensitivity and feeling to recognize women's insecurity or indecisiveness, which could then reveal a RCA context. They ensure that they meet with women alone to explore the situation and offer support. They focus on creating a safe space to reassure women, particularly about issues of confidentiality. This echoes the expectations of women experiencing intimate partner violence, as documented in a recent qualitative data synthesis by Korab-Chandler and colleagues [26], to be reassured. Women fear for their safety and the safety of their children, which makes it all the more important to establish a trusting relationship, including active and empathetic listening [26] and care-based emotional support [27]. Finally, health professionals inform women of available services and share available contraceptive methods to prevent future unintended pregnancies [25, 27]. These findings can be related to the guidelines for frontline response to violence against women developed by WHO [64]. The LIVES model proposes the following five steps: Listen, Inquire about needs and concerns, Validate, Reinforce safety, and Support. These steps would provide optimal support for women experiencing intimate partner violence. The CARE model [27] also provides relevant guidelines for supporting women experiencing intimate partner violence. This model is divided into four components: Choice and Control, Action and Advocacy, Recognition and Understanding, and Emotional Connection. In Quebec (Canada), an intervention model based on the strengths-based care model [28] has been developed for nurses to support their practice in domestic violence [29].

While these models place the human qualities of SPs at the center of their guidelines, it seems relevant to transpose an adaptation of these models to the specific context of RCA. It is also important to tailor intervention strategies to the population served. A culturally sensitive approach is needed to support women who are not part of the dominant groups in a society in terms of, for example, ethno-cultural background, sexual orientation, and gender identity. In this regard, Tarzia and colleagues [30] document professional practices of SPs in Australia that take into account the systematic barriers that women from minority ethnic backgrounds may face. Indeed, SPs cite the complexity of RCA coupled with immigration issues (visa cancellation, access to services) and financial instability (inability to work without a visa or receive government financial aid). It remains unclear how women are asked to identify experienced RCA, and few concrete examples are given. In addition, many women may take time before disclosing the RCA they have experienced for various reasons [31]. In Quebec, little information and tools are currently available on how to accompany and support women. The development of intervention practices specific to the context of RTA appears necessary to best accompany people who have experienced RCA. This would allow a variety of SPs to take hold of these guidelines in order to integrate them into their professional environment.

In Canada, where this problem has yet received limited research attention [32, 33], studies have not documented knowledge of RCA in SPs or their challenges in intervening with the victims. This knowledge is critical for optimizing or offering specific RCA intervention strategies in institutions and community organizations that provide reproductive health services or violence counselling. To document this social and health issue, we conducted a qualitative action research with a community organization, the Fédération du Québec pour le planning des naissances (FQPN) (Quebec federation for family planning) and l’Institut national de santé publique du Québec (INSPQ) (Quebec national public health institute).

In this article, we describe the two consecutive phases of this participative action research project targeting RCA. First, we will present the results of a qualitative research process aimed at better understanding clinical practices as well as the barriers and facilitators encountered by SPs regarding RCA, as well as their information and training needs. Next, we will briefly outline the process of collaboratively developing intervention tools for SPs to mitigate identified barriers and provide useful knowledge and guidance. The brochures will be available for consultation in the Additional files 1, 2, 3.

## First phase: needs assessment

To identify intervention-related issues from an interdisciplinary perspective, we held focus groups in which we encouraged SPs to share their professional experiences about RCA and describe their information and training needs. This data collection method allows discovering new content to enrich the understanding of clinical issues [34]. Moreover, it provides an opportunity for peer learning and networking, which can contribute considerably to improved preventive approaches. This study was approved by the human research ethics committee of the principal researcher’s university (3661_e_2019). The research team comprised the two first authors who jointly led all the focus groups. The two other authors participated actively in the study advisory committee and the writing of the results and discussion for this article. Based on the new knowledge generated by the focus groups, intervention tools were developed and improved by also reviewing the best practices identified in the grey and scientific literature, existing intervention tools, feedback from focus group participants, the study advisory committee and experts in the field of reproductive health and gender-based violence.

## Methods

### Recruitment

The inclusion criteria for the focus group participants were twofold: participants had to (1) work in in the field of reproductive health or violence against women; and (2) have at least two years of experience with this clientele. Participants were recruited via professional networks for organizations and groups specializing in sexual and reproductive health and violence and via recruitment ads posted on social media networking sites.

### Data collection

To facilitate the data collection, we developed a semi-structured focus group guide based on the RCA literature and the expertise of the study advisory committee. The discussion topics addressed RCA situations that the SPs dealt with at work, their perceptions of this issue, the interventions they used when RCA was suspected or identified, their degree of comfort when dealing with this issue, and their needs in terms of training. Open questions were used to encourage the participants to share their opinions and develop new ideas.

We held five focus groups. In addition, we held one individual interview with a participant who was unavailable for the scheduled focus groups. The groups comprised from 3 to 14 participants and the discussions lasted from 60 to 90 min. Data were collected from November to December 2019 in various regions of the province of Québec (Canada). All participants signed a consent form and a confidentiality agreement.

### Analysis

All data were transcribed verbatim and entered in NVivo 12 for thematic analysis in accordance with the study objectives [35]. Inductive coding, through NVivo, was first used to classify the data into codes. Through thematic analysis, we identified the relationships between the codes to develop a comprehensive understand of the SPs perspectives on RCA. This then allowed the identification of the main themes and the subthemes which generate meaning [35]. The preliminary results were submitted to the project partners and the research team for comments. The results were then reformulated to address the clarifying comments of the partners and the research team. Mixed coding was initially performed on all data. Results were then submitted to the study advisory committee for discussion. This enabled new insights and diverse approaches to the analysis results and paved the way for identifying relevant themes. All professional information (e.g., job title, workplace) was retained in the transcripts to enable contextualization and to aid the analysis. However, all confidential information has been removed from the transcripts that are presented in this article.

### Participants

We held discussions with 31 SPs working in six different professional settings: community health centres specializing in sexual and reproductive health, medical facilities, abortion clinics, and community organizations specializing in violence against women. The participants had a diversity of expertise in RCA and intimate partner violence (IPV). Nearly three-quarters of the participants work in community health centers that specialize in sexual and reproductive health, while some participants work in a community organization that specializes in domestic violence and some work in the medical field or in an abortion clinic. They had several years of seniority at the same organization, ranging from 2 to 23 years. All participants identified as cisgender women.

## Results

In the focus groups, some SPs immediately acknowledged the presence of RCA behaviors in women and individuals seen in consultation, while others did so further along in the focus groups as they became more aware of the various forms of RCA. Globally, there were no major differences in the responses given by participants according to their work affiliations. It should be noted, however, that the context of domestic violence advocates differs from that of other service providers, as individuals who come to them do so specifically to discuss the violence they have experienced, rather than to address sexual or reproductive health issues. The most frequently identified situations were when a violent partner either forced an abortion or prevented access to one. Some participants had met women who were subjected to physical violence when they were pregnant, resulting in miscarriage. They also reported situations involving contraceptive control and sabotage, mainly non-consensual condom removal or some form of lying about condom use. The SPs also encountered women whose partner, contrary to what had been agreed, failed to pull out before ejaculating, lied about having a vasectomy, or prevented the woman from accessing contraception.

Faced with these situations, the SPs used various strategies to respond to the women’s needs and provide them with support.

### Intervening in cases of reproductive coercion and abuse

We identified the main elements of the interventions used across the workplace settings. These may be broken down into two phases. First, the SPs explored the situation that drove the woman to consult for services their organizations offer. Generally, this meant looking at overall health and the relational context. Next, they attempted to identify and discuss RCA situations.

#### Observation and sensitivity: looking for indications of the relational context and the client’s needs

The SPs said that they began by asking their clients about their life context in general, their relationship, and their overall health. They paid particular attention to the clients’ general state of health, emotional as well as physical. They believed that the client’s emotional state was one of the main indicators of RCA. More precisely, based on their professional experience, noticeable anxiety sent a signal that the situation needed further exploration. The anxiety could manifest as concerns about the length of the appointment, about marks left on the body after a medical exam or treatment, or about the potential consequences of opening a medical file:

When it takes a long time, then the woman worries about when she’ll be leaving, because she didn’t tell her partner that she was coming for an abortion. Or, […] when we’re going to put in a catheter, then she doesn’t want there to be any marks. (FG4)

Other possible signs of RCA were mood disorders, somatic disorders, and painful sex. Such problems alerted the SPs to delve deeper. Another indication was the presence of bruises in the physical or gynecological exam.

One woman who had bruises, […] really a lot of bruises, you know, that I wondered about. And I was happy to see that in the discussion with the doctor who was here that day, she made a point of questioning the woman about them. I think that we shouldn’t shy away, you know. It’s not easy, but it’s necessary. […] It can be an opportunity to pursue the discussion, in fact. (FG4)

The quality of the relationship was another important matter that the participants emphasized. They said that the partner’s attitude, if he accompanied the woman to the appointment, could be revealing about the power relationship with the intimate partner. For example, they recounted cases where the partner controlled what information was disclosed during the appointment by speaking on the woman’s behalf, or else by getting impatient or angry.

As soon as they walk in, we usually see it. When a woman brings her partner, then it’s the partner who takes the lead in the discussion. So, […] he’s the one who’s going to ask the questions. He’s the one who’s come to see us. (FG4)

Accordingly, the SPs had developed clinical practices that were sensitive to indications of potential RCA. These were based on the feelings and skills they had honed over their professional career. Whenever they suspected violence, they proceeded to a more targeted detection of RCA.

#### Detecting the presence of violence in the relationship: questioning and remaining vigilant

Some health organizations had developed sets of questions about domestic violence that they routinely asked any women who came in for a consultation. One participant said that these questions set up a climate of non-judgmental listening that made it easier for the women to talk about past or ongoing violence.

In my practice, we do a screening with all the women. To find out if they’ve experienced violence in the past, whether it’s psychological, physical, or sexual. And then, we also tell them a lot that pregnancy and childbirth, these are tipping points when things could erupt, that might be forgotten later, and that they shouldn’t be embarrassed to tell us about them afterward, either. (FG1)

Participants working in other organizations preferred to ask targeted questions about the relationship to identify RCA. The objectives included finding out the length of time the woman had been in a couple with her partner, if there was a significant age difference between them, if they were planning to have children, and if they sometimes argued about contraception.

We would ask, “How long have you been with your partner?” “Are you using some form of contraception?” And without laying a guilt trip, we try to understand why it didn’t work: “So, what made the contraception fail?” (FG2)

Other questions targeted notions of control, freedom of choice in matters of contraception and reproduction, and the various available options for reproductive health. Although the women might give evasive answers, they sometimes disclosed their partner’s controlling behavior.

You know, they don’t say, “I experience domestic violence.” Instead they say, “Well, he doesn’t let me do this or that,” or, “He doesn’t want me to use an IUD, so he […] made me give it to him so that I wouldn’t have any contraception. (FG1)

The participants had adopted different intervention styles, according to their comfort level, personality, training, and years of experience. Some asked more direct questions. Others approached the subject more gently, especially when it came to contraceptive issues they discerned in their clients’ discourses. This helped them identify the presence of control in the relationship or unequal relations between the partners. Generally, the participants felt that this identification process, either systematic or not, worked well to detect RCA situations.

### Supporting women who are victims of RCA: guiding actions according to perceived needs

After exploring the issue and more specifically identifying the client’s situation, the SPs guided their interventions according to four major needs: safety, psychological support, harm-reduction strategies, and referral.

#### Ensuring that the women are safe

Once the RCA was disclosed, the SPs began with an assessment of the women’s safety. For example, they would ask them if they had access to a support network or a temporary shelter.

"We have to check if they are safe, if they have a support network and resources. [... If they are safe, my mandate is to listen to them and provide resources if needed. They are resources for violence. (FG2)

As needed, the SPs could refer the women to organizations that provided immediate and secure assistance (e.g., emergency shelters).

#### Believing the women and validating them

Because their clients often felt ashamed and guilty about the violence that was done to them, the SPs tried to reframe these emotions by asserting that it is not their fault while offering an empathetic ear. For example, one participant regularly told her clients, “You know, you’re not responsible, and you’re not alone” (M-FG2). Depending on the services that the organization provided, they suggest psychosocial support:

We can invite them to come back to the clinic. Because, you know, often, it’s a long day for them [having an abortion]. “If you’re not sure today, you know, you have a file with us now. You can come back to the clinic whenever you want. It’s completely confidential here.” (FG4)

#### Proposing protective strategies to reduce harmful behavior

Interventions that were designed to reduce the risk of violence, in a harm-reduction perspective, were mostly reported by participants who worked at reproductive health organizations compared to other settings. They described these interventions as protective strategies. For instance, some women do not necessarily want to end the relationship with the perpetrator. For this reason, they need tips on how to maintain control over their contraceptive and reproductive choices to reduce the risk of violent reactions from their partners. In these cases, the SPs could inform the women about what happens when there is a miscarriage. The women could use this information later to explain why her pregnancy ended without revealing that she had an abortion.

At times, we have to give them tips. Because when a woman comes in secret, to get an abortion, we can teach her some tricks. Like what to tell her partner. […] You can say that you had a miscarriage and that you came to the clinic for a checkup, and things like that. (FG4)

It could also be a matter of choosing a concealable contraceptive method like an injectable contraceptive, or they might suggest cutting the strings of the IUD very short so that her partner wouldn’t feel it.

#### Ensuring continuity of services after the consultation via referral

Most of the participants referred victims of RCA to an appropriate resource. Of these, the most common were organizations specializing in gender-based violence: they provided a listening ear, counselling, and a place to stay. Next came public organizations (i.e., state-funded clinics that provided psychosocial support) and professionals in private practice (i.e., sexologist, psychologist). The SPs described how they supported and guided the women on this journey:

Usually, we take steps on their behalf if they need it. We establish a connection. We might act as go-between too, with different people, all depending on […] on the situation as well, the woman’s case. So we don’t just give them a phone number and then leave them to it. (FG1)

### Barriers and facilitators for optimizing intervention practices

Although the SPs working in reproductive health could identify the presence of RCA, they sometimes felt limited at the clinical level due to workload, organizational shortcomings, communication problems with clients, and lack of training. That said, they believed that the seriousness they gave to the issue and their confidence in their intervention strategies sometimes helped them overcome these limitations.

#### Restraints on intervention practices

*Interventions were limited by time constraints.* Almost all the participants blamed lack of time as the main barrier to RCA intervention. To meet the demand and provide services to a maximum number of clients, appointments had to be closely scheduled. This left little time to talk things over with the women who came for help:

It’s hard to tell, then. You spend 15 or 20 minutes with the woman. You can have your doubts, but, […] So, [silence] […] For me, I’m calling it like it is, but I find that our scope is limited. But I think that it’s probably behind this little door here, [it’s] the only time when they’re willing to do it, if they feel they’re up to it, where they have a space to do it. (FG4)

Considering that many of the reproductive health services for which women seek care require only one medical appointment, some SPs were rarely able to provide the women with follow-up. Sometimes they didn’t dare begin a discussion about violence because they were afraid that they wouldn’t have time to approach it properly.

But the problem, sometimes, is that we, here, at the [abortion] clinic, we have one problem to fix, and it’s an unwanted pregnancy. So that means that usually, we’re going to focus on that. You know, the forms of violence that we hear about, well […] we give them tools, but you know, we cannot do any follow-up and all that. (FG5)

*Poorly equipped and insufficient facilities.* Lack of resources impacted how the interventions were carried out. For example, the participants mentioned a lack of adequate physical spaces at their organizations: many rooms were not soundproofed, which could inhibit the women from disclosing any violence done to them, particularly if their partner was waiting next door.

You know, a lot of women come in here with their partners, and if they’re accompanied, even if they’re in that office there [pointing to a room], the partner is sitting right over there [pointing to an adjacent room]. So they don’t dare talk about any violence that they experienced. You know, I mean. […] It’s not very private, so the women don’t want to take the chance. (FG4)

According to the SPs, these material factors impacted the women’s willingness to disclose their stories.

*Language and cultural barriers.* Many participants felt that perceived language and cultural barriers could prevent some women from seeking intervention. They described how hard it was to intervene with women who spoke neither English nor French in cases where the SPs only speak these two languages. While French is the official language in Québec, some organizations can offer services in different languages in addition to English and French, the two official languages of Canada. Nevertheless, most organizations lacked the required funds to hire interpreters or translate written material. In addition, some participants reported that the interpreters sometimes knew the women or belonged to their community, which raised confidentiality issues.

Sometimes only the partner speaks French or English and is the one to act as interpreter. These situations limit the scope of the discussions, particularly when it comes to relational matters and prevent the SPs from investigating into issues of violence. It also prevents them from sharing harm-reduction strategies to women. Some SPs added that it is difficult to explain, in these conditions, the contraceptive options, reproductive health processes, and the notion of sexual consent to the woman: “For sure, the language barrier […] You know, it’s hard for us to […], for me, to properly explain their options in terms of contraception, in terms of sexual consent too.” (FG1).

Cultural distance was an issue for all the organizations. The participants recounted how some women were hesitant to talk about sensitive and personal subjects, and they felt that this could be linked to cultural or religious values. As one HP explained, “If she refuses to talk because of her personal values, […] If she says, ‘I don’t want to talk about that,’ I’ll leave the door open, but I can’t force her” (FG3). Others found that women who had recently left their country where abortion was illegal tended to associate abortion with fear and danger. They were distrustful, and they were afraid that they would be refused the abortion if they didn’t give the correct responses.

In some countries, an abortion is practically impossible to get, so when they come here, they’re really scared of saying something that could prevent them from getting the abortion, so they say nothing. The new immigrants are so afraid to be refused access to an abortion that they give quick answers, and sometimes false ones, to health-related questions. (EI1)

*Lack of training in social intervention.* The participants stressed that it was hard to manage RCA situations properly due to lack of training in best intervention practices. One participant explained that her university training program barely addressed psychosocial support. Therefore, she felt that she could offer only limited support when an RCA situation arose:

I’m a nurse. I’m not a sexologist. The counselling and therapeutic relationship, I learned a little bit about it, there, but we only spent a few hours on that at school. So for this kind of thing, sometimes, I feel sort of limited (FG4).

Another participant added:

I’d like to be better prepared to help them move forward, even if it’s ever so little, so that they can go and find really good professional help, but I don’t even feel like I can be an effective go-between (FG4).

#### Facilitators for clinical practices

Despite these barriers to intervention, the SPs identified elements that enabled them to optimize their clinical practices. The importance they gave to RCA intervention, their accumulated knowledge of the issue, and their professional experience made them feel confident about the quality of the support they provided to the women they met.

*Caring about the wellbeing and the security of their clients.* All the participants believed that it was very important to provide their clients with a safe environment, and they felt it was their responsibility to intervene in cases of violence.

It’s something that’s primordial, that’s […] it’s basic. You know, that the woman feels safe, feels okay. So, for me, it goes without saying that it’s super important. To bring up these subjects, so that she feels comfortable enough to open up, or not. You know, even if they hold back, if she’s not ready to open up, at least she knows there’s somebody here for her, if ever she feels like talking about it. (FG1)

For another participant, failure to disclose the violence did not equal a failed intervention. Instead, she viewed it as the initiation of a process of recognition for the woman, plus the knowledge that she can turn to the organization whenever she’s ready to talk about her situation.

It’s super important for her personal safety, and so yes, she’s going to know that there’s a possibility to talk about it. That alone, it’s already a successful intervention, even if the person doesn’t get away from her relationship, or his unhealthy behavior. There’s at least the possibility there of the ability to speak up when she’s ready. (FG1)

Generally, the importance that the participants gave to the issue motivated them to propose intervention strategies.

*Confidence in their intervention strategies.* The participants said that the knowledge and understanding they had acquired over time had given them an awareness of violent situations. This knowledge gave them confidence about their interventions and their usefulness. Moreover, it helped them develop effective ways to intervene:

I would say that it’s more confidence, the years of experience. Whereas, before, sometimes, you’d try out three or four ways, and then you don’t even end up asking your question […] Sometimes, I tell myself to get straight to the point. Sometimes, it could be the best way. (FG5)

Thus, they refined their interventions according to the outcomes they obtained with their clients. When they saw that some methods and approaches were more effective than others at drawing out testimonies of violence, it helped build their confidence as HP-SPs.

## Second phase: literature review of best practices and development of intervention tools

The intervention tools were developed based on the results of the focus groups and a literature review of best practices in intervention. The results of the qualitative data collection phase of this project have several clinical implications that were useful in the subsequent phase. Foremost, the participants expressed the need for training in RCA so they could better detect and adequately support victimized women. To meet this need, information and training in RCA are required to optimize the attitudes, knowledge, and skills of SPs. By conducting a literature review in the grey and scientific literature, we identified best practices for intervention in RCA [36], domestic violence [37–41] and domestic homicide risk identification [42]. We also identified the preferred approaches to intervention on these issues which are the trauma-sensitive care approach and cultural safety [43, 44].

It has been demonstrated that brief, specific interventions provided at family planning clinics can reduce violence against women, RCA, and unplanned pregnancies in women aged 16 to 29 years [45]. For example, the Addressing Reproductive Coercion in Health Settings (ARCHES) protocol consists of a brief, three-hour training program for clinical staff. It addresses IPV and RCA, methods to encourage women to disclose such behavior, and appropriate counselling and referrals. The aim is to reduce harmful behavior and the unintended consequences. The results of a cluster randomized controlled trial demonstrated the feasibility and effectiveness of this approach [45]. This intervention was recently adapted to the Kenyan context in community health clinics in Nairobi (Kenya) [46]. Moreover, the American organization Futures without violence were pioneer in this aspect and developed a Guide for Obstetrics, Gynecology, and reproductive Health care Settings intitled “Addressing Intimate Partner Violence, Reproductive and Sexual Coercion”, as well as a safety card designed specifically for women [36]. The development of the two intervention tools was inspired by this earlier work to help implement best practices.

For the development of the intervention guide for SPs, the objectives were to (1) inform about RCA, (2) propose best practices for intervention on RCA and violence against women, (3) share available resources and training on RCA and violence against women. Following the participant’s recommendations, we developed a second booklet aimed at women and individuals who seek sexual and reproductive health services. It is a reflective tool that allows people to think about their intimate relationship and the contraceptive and reproductive choices they may take in that relationship. The intervention tools are developed with a feminist, culturally safe and trauma sensitive approach [47–49]. Cultural safety is essential to improve interventions that target different groups, such as people who have immigrated, racialized communities, and individuals with diverse sexual and gender orientations, low income [50], or disabilities [51].

In an iterative process, the content of these two intervention tools was adjusted and validated by experts in the field, the study advisory committee and some of the focus group participants. Additional sections were added, such as indicators to look for during a consultation, following feedbacks from SPs. The brochure for women and non-binary people seeking reproductive health services has been translated into English, as a portion of Quebec residents are English-speaking, and English may be the language of use for many First Nation, Métis and Inuit people as well as many immigrants or refugees. Following a graphic layout to make it accessible to people with limited literacy levels, the booklet was submitted to the general population for feedback. A mail-out campaign distributed printed copies to over 15 organizations, while a digital distribution has enabled us to reach more than 50 health and intervention settings to disseminate the online version of the tools. Presentations and discussion workshops were held in different settings, at the national and local levels, to present the tools developed, and more broadly the issue of reproductive coercion. The intervention guide for SPs and the reflective booklet for women and individuals consulting for health care services are available online, on the community partner’s website (www.fqpn.qc.ca). (Readers can consult them in the Additional files).

## Discussion

This article first documents the clinical intervention practices of service providers in Québec (Canada) in relation to reproductive coercion and abuse, and then briefly reviews the process of developing and validating the information and awareness tools for the target audience. To our knowledge, this is the first Canadian study to explore the knowledge, attitudes, and skills of SPs in this area. Globally, the participants were aware of the issue of violence against women and RCA and did not adopt an attitude of denial of the possibility that many of the people who consulted them were experiencing such situations. Some of them were able to describe the counselling and support they provided in these situations. Concurring with other studies, the SPs said that they were able to talk openly about RCA and associated forms of violence [52]. Moreover, in contrast to the quantitative study by McGirr and colleagues [52], the SPs in our study reported that they regularly explored relational contexts with the women who came for consultations, which could have helped them detect RCA behavior. This difference could explain in part the potentially greater knowledge and experience of RCA situations in our participants.

Women and non-binary individuals may seek SPs because their sexual and reproductive health had been threatened, particularly by RCA behavior. Consistent with the findings of other studies, the SPs recognized that RCA impeded the women’s reproductive autonomy, and they considered it a form of violence against women [24, 36]. Accordingly, when women sought health or psychosocial services, all the SPs began by exploring the overall situation. This enabled them to detect potential controlling or violent behavior. It is probable that the feminist interventions that the organizations we met had adopted, or that many of the participants used, gave them a deeper understanding of the continuum of violence to which women are subjected. Consequently, they were able to provide different types of interventions. The overriding aim was to minimize harmful behavior, which is a common strategy used in this field according to other studies [12]. They offered interventions that were guided by the principles of safety, psychosocial support, minimization of harmful behavior, and referral according to the client’s needs. These interventions were meant to support women who were dealing with not only RCA, but also psychological, physical, economic, and sexual violence [53].

All the workplace settings featured barriers that limited the scope of their interventions. Lack of time was the main challenge, which prevented the SPs from providing their clients with adequate care and support without rushing the process, as reported in other studies [25, 54]. This made some SPs feel powerless, or at least limited in their power to intervene properly within the allotted time slot for consultations. This feeling can lead to inaction [55]. Lack of private spaces, the limited number of services offered, and the difficulty of establishing follow-up were further organizational limitations. Language and cultural barriers posed additional challenges, mostly in the urban settings, and especially when appropriate interpreters were unavailable. This situation calls for concrete actions, even more so because studies show higher RCA prevalence rates for racialized and immigrant populations [4, 56, 57]. To ensure accessibility to services and interventions for clients who are migrants or from non-majority cultures, it is necessary to be mindful of cultural safety [58, 59]. Finally, the SPs working at all the organizations stressed that they lacked training, in line with other studies in this area [52, 55]. They believed that the training should integrate a feminist perspective in which RCA is situated along a continuum of sexual violence against women [60]. Training on violence against women and specifically on RCA should be offered to SPs, in accordance with the recommendations made by Sprague and colleagues [61] to adopt an interdisciplinary approach to address violence. These trainings should also be evaluated to ensure that they are effective and that they incorporate the perspectives of the people who access the services [61]. Furthermore, RCA should be socially problematized. This would broaden the individual perspective to accommodate a concerted and interdisciplinary response that considers prevention as well as intervention. All the SPs described factors that facilitated their clinical practices. These were largely related to individual characteristics: the importance they placed on discussing issues of violence and the confidence in their own skills they had acquired with years of experience, concurring with other studies [24, 62].

### Limitations

This study includes certain limitations. First, the convenience sample comprised individuals who were already aware of violence against women or RCA and reproductive health issues. All the participants were willing to discuss this health and social issue and describe how their organizations responded. Most of the organizations had adopted a feminist approach to intervention from a caring, trauma-sensitive perspective. A more diversified sample in terms of clinical expertise in RCA would presumably reveal more shortcomings in the SPs’ intervention practices. Second, some focus groups were composed of members of a same work team. Greater diversity of settings across the focus groups could have allowed the expression of new ideas, and the participants would have been exposed to other professional realities. It is possible that these discussions would have enriched and reshaped their views on clinical practices for RCA.

## Conclusion

Despite the recent widespread recognition of the RCA issue in research and professional communities, much remains to do so that victims of RCA have full access to the services they need. The barriers described by the SPs should be addressed and rectified to allow optimizing professional interventions in line with feminist, trauma sensitivity and cultural safety approaches [12, 59]. We believe that intervention tools can help to address part of this gap by providing SPs with strategies to enhance their intervention practices related to RCA. However, further work is needed to produce a concerted response to RCA, including the development of prevention strategies tailored for diverse populations, regulations, law enforcement, and important social awareness work on gender-based violence. Finally, RCA calls for an interdisciplinary response that combines expertise in law, public health, clinical health care, and social work. This response should involve not only SPs, but also all the health and psychosocial organizations that provide services to individuals who are or have the capacity to become pregnant.

## Supplementary Information

**Additional file 1.** Intervention Guide for SPs (French).

**Additional file 2.** Booklet (French).

**Additional file 3.** Booklet (English).

## Data Availability

The booklets are supplied in Additional materials section and are also accessible free of charge on the project partner's web site: www.fqpn.qc.ca
