# Supplementary material for: Canadian service providers' perspectives on reproductive coercion and abuse: a participatory action research to address their needs and support their actions
Source: Reprod Health. 2023 Jun 30;20:100. doi: 10.1186/s12978-023-01640-w (PMC10311789; doi:10.1186/s12978-023-01640-w)
Supplement: Supplementary file 1 — Additional file 1. Intervention Guide for SPs (French). [file 12978_2023_1640_MOESM1_ESM.pdf]

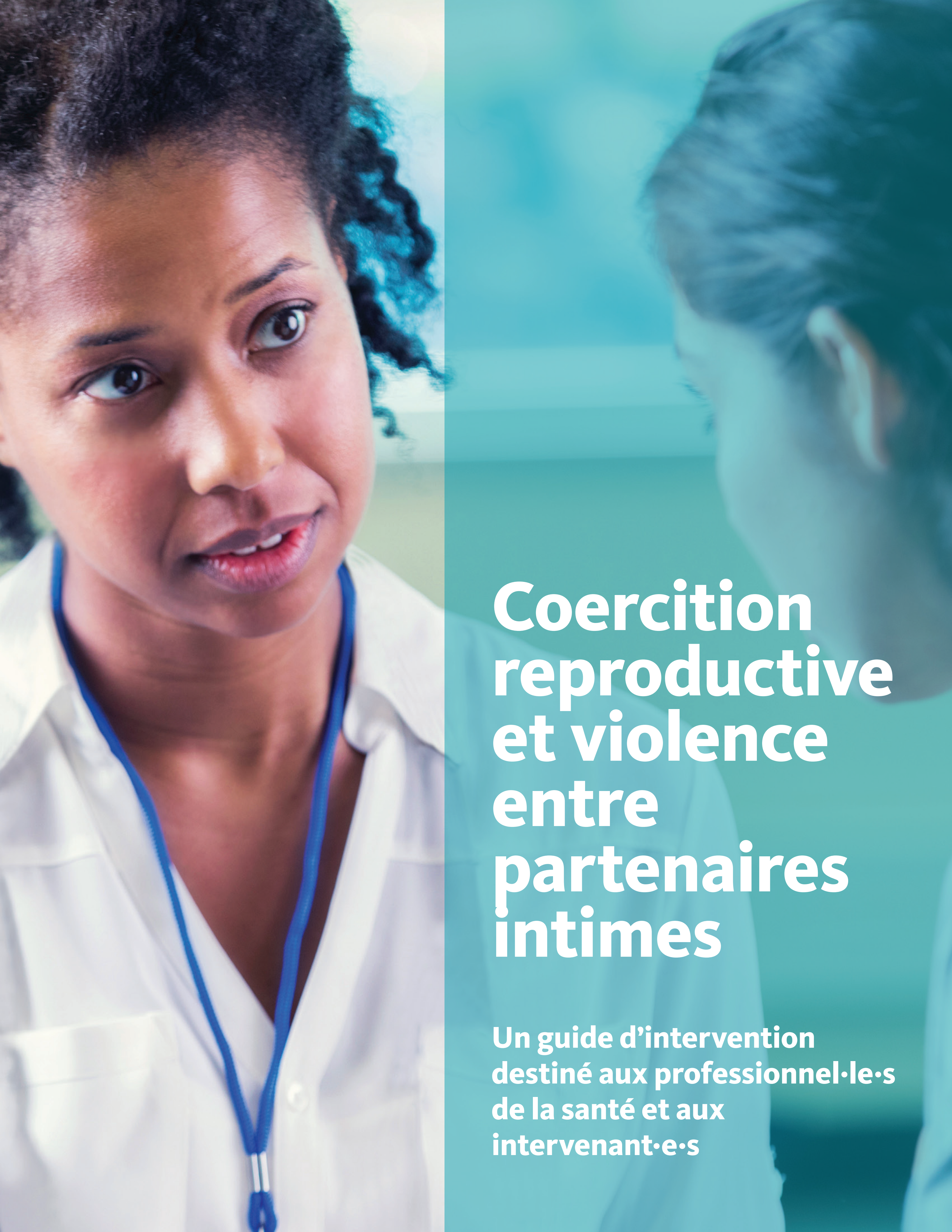

Coercition reproductive et violence entre partenaires intimes

Un guide d'intervention destiné aux professionnel·le·s de la santé et aux intervenant·e·s

Ce projet a été réalisé sous la direction de :

SYLVIE LÉVESQUE, Ph.D.
Professeure, Département de sexologie,
Université du Québec à Montréal
levesque.sylvie@uqam.ca

Avec le soutien de :

CATHERINE ROUSSEAU, M.A. sexologie
Agente de recherche,
Université du Québec à Montréal

Organismes partenaires

Fédération du Québec pour le planning
des naissances (FQPN)

Institut national de santé publique du Québec (INSPQ)

Nous remercions sincèrement les intervenant·es, professionnel·les et les organismes qui ont participé aux groupes de discussion, à la révision du document et/ou à sa validation.

JULIE LAFOREST
Chef d'équipe, Sécurité, prévention de la violence
et des traumatismes, INSPQ

CAROLINE ROBITAILLE
Coordonnatrice
Équipe de recherche Violence conjugale,
acteurs en contexte et pratiques novatrices

LOUISE HAMELIN BRABANT
Professeure associée-titulaire retraitée
Faculté des sciences infirmières
Université Laval

JULIE ROBILLARD
Co-coordonnatrice, FQPN

LAURENCE RAYNAULT-RIOUX
Chargée de projet ACSEXE+, FQPN

Centre de santé des femmes de Montréal

Clinique des femmes de l'Outaouais

SOS Grossesse

Violence Info

Et les étudiantes du Laboratoire de recherche
sur la santé reproductive et les violences :
CAROLE BOULEBSOL et **ALEXANDRA TOUPIN**

Révision linguistique

VÉRONIQUE PHILIBERT
Révision Œil Félin

Conception graphique et mise en page

JULYE MAYNARD

Référence suggérée :

Lévesque, S. et Rousseau, C. (2021). *Coercition reproductive et violence entre partenaires intimes. Un guide d'intervention destiné aux professionne·le·s de la santé et aux intervenant·e·s.*
Montréal : Université du Québec à Montréal.

Ce projet a été soutenu financièrement par le Secrétariat à la condition féminine dans le cadre de l'appel de projets *Sensibilisation en matière de violence conjugale et de violence sexuelles 2018-2019.*

Dépôt légal
Bibliothèque et Archives nationales du Québec, 2021
ISBN : 978-2-9818518-1-9

Table des matières

POURQUOI CE GUIDE ? POURQUOI VOUS EST-IL DESTINÉ ? 5

COMMENT UTILISER CE DOCUMENT ? 9

Objectifs du guide 9
Descriptif du contenu 9

SURVOL DE LA PROBLÉMATIQUE 10

Qu'est-ce que la coercition reproductive ? 10
Quelles formes peut prendre la coercition reproductive ? 11
Quelles sont les répercussions de la coercition reproductive ? 15
Quels contextes peuvent augmenter la vulnérabilité ? 17
Indicateurs à surveiller lors d'une consultation 18

PISTES POUR DES PRATIQUES D'INTERVENTION ADAPTÉES 19

Approche de soins sensibles aux traumas 19
Sécurisation culturelle 21

STRATÉGIES D'INTERVENTION POUR LE REPÉRAGE DE LA COERCITION REPRODUCTIVE 22

Accueillir 23
Identifier 24
Soutenir 30
Diriger 33

EXEMPLES D'INTERVENTIONS BRÈVES 35

POUR EN SAVOIR PLUS 38

La production de ce fascicule et la brochure destinée aux femmes et aux personnes concernées est le produit d'un travail fait en collaboration avec des organismes et institutions spécialisés en violence conjugale, en santé reproductive et en santé publique, et des professionnel·le·s et intervenant·e·s rencontré·e·s sur le terrain.

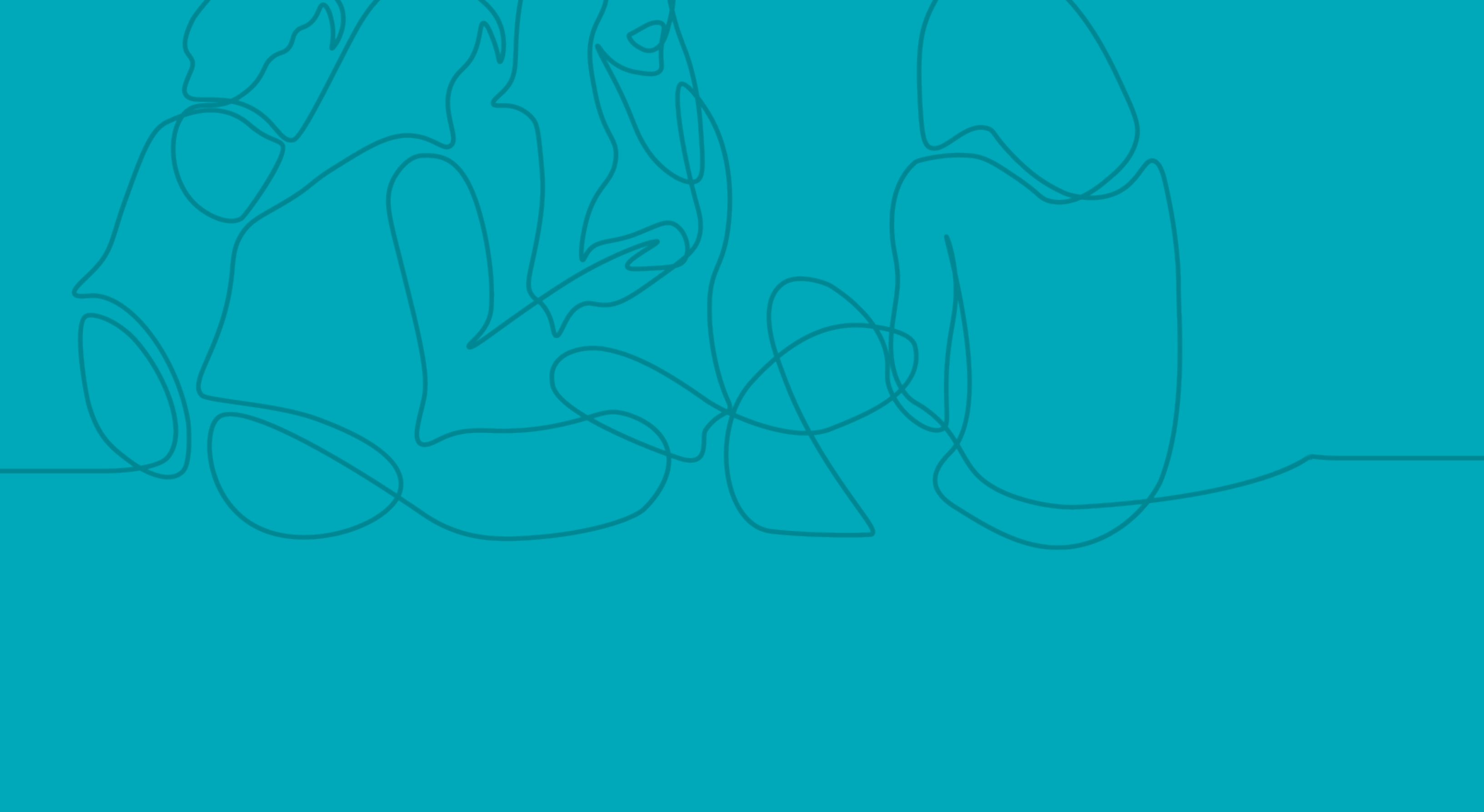

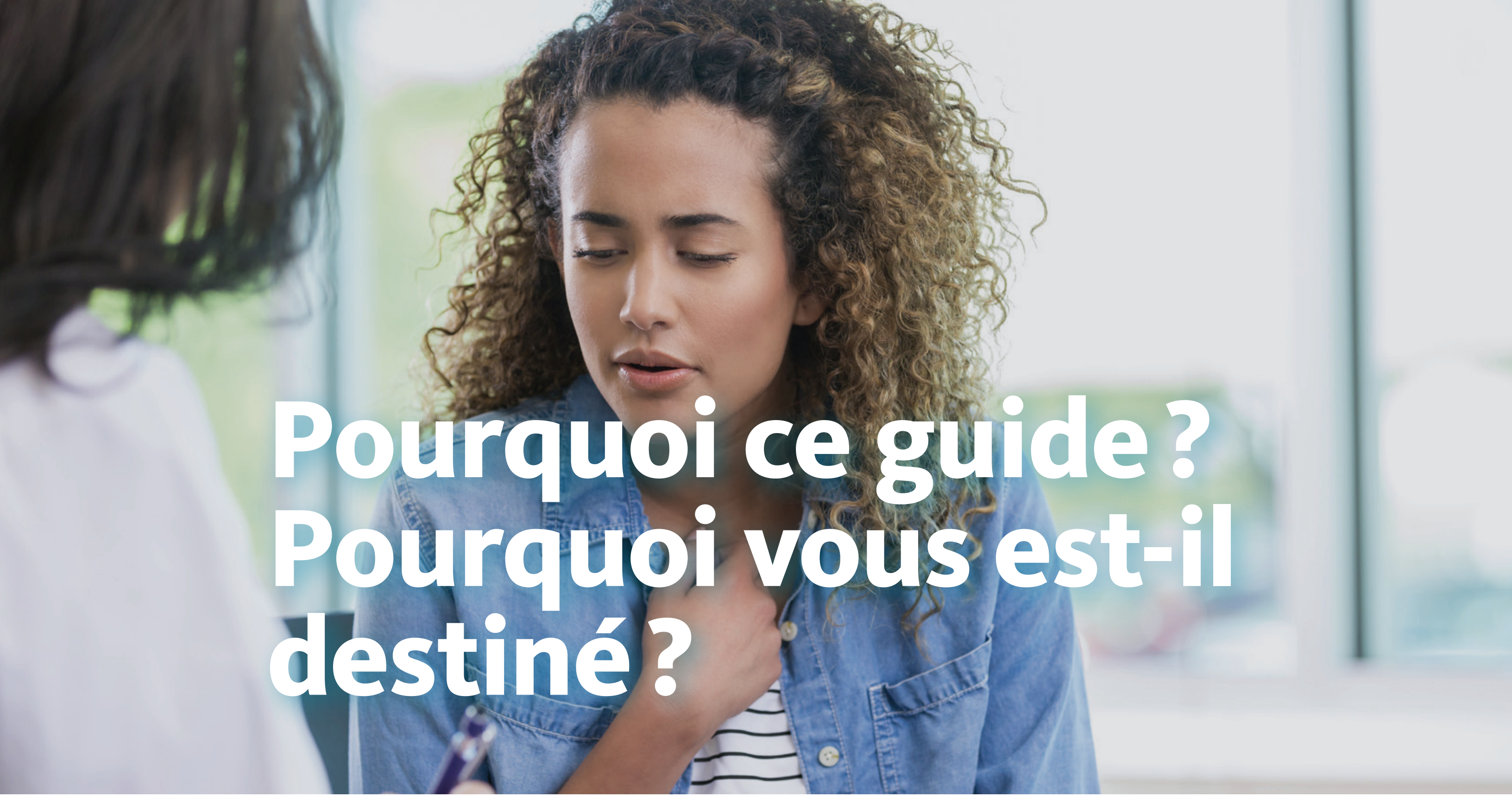

Pourquoi ce guide ? Pourquoi vous est-il destiné ?

La violence faite aux femmes est une problématique sociale et de santé publique importante. Elle entraîne de nombreuses conséquences pour celles qui la subissent.

Cette violence peut survenir entre partenaires intimes, que ce soit dans un contexte amoureux ou sexuel, et prendre différentes formes. Lorsqu'elle cible la reproduction, nous parlons de coercition reproductive.

La coercition reproductive réfère à des comportements qui interfèrent avec la contraception et la planification des naissances, tout en réduisant l'autonomie des femmes quant à leurs choix reproductifs[1].

Celle-ci se manifeste de différentes façons (pour plus d'information sur les manifestations, consultez la section *Quelles formes peut prendre la coercition reproductive*). Par exemple, les comportements peuvent viser à empêcher ou à saboter la prise de contraception, à mettre de la pression ou à imposer une grossesse ou un avortement. Ces comportements sont posés sans égard aux intentions reproductives des femmes concernées.

Les femmes et les personnes qui vivent de la coercition reproductive ont davantage recours aux services de santé que celles qui n'en vivent pas[2]. Cela représente une occasion d'intervention pour les professionnel·le·s de la santé et des services sociaux, ainsi que les intervenant·e·s qui travaillent dans les milieux de la santé sexuelle et reproductive ou dans les organismes spécialisés en violence faite aux femmes.

Considérant :

- l'importance des répercussions de la coercition reproductive sur la santé sexuelle et reproductive,
- la gravité des impacts négatifs de la coercition reproductive sur la santé mentale et physique,
- les demandes de services associés à ces conséquences (p. ex. : dépistage d'infections transmissibles sexuellement et par le sang (ITSS), contraception d'urgence, etc.),

il est nécessaire de s'informer sur la violence entre partenaires intimes et la coercition reproductive afin de mieux soutenir les femmes et les personnes qui en sont victimes.

« Les femmes qui me consultent ne vivent pas de violence... »

Il est possible d'avoir l'impression que la violence envers les femmes est marginale et ne touche qu'une petite proportion d'entre elles. Détrompons-nous : la violence faite aux femmes est une réalité fréquente survenant dans tous les milieux socioéconomiques et aux différents âges de la vie. La violence faite aux femmes ne se limite pas à la violence physique, mais comprend aussi la violence psychologique, la violence sexuelle et la violence économique, pour ne nommer que celles-ci.

Voici des données permettant d'illustrer l'ampleur de cette situation **:**

- En Amérique du Nord, **1 femme sur 5** vivra de la violence de la part d'un partenaire au cours de sa vie[3].
- En 2014, **1 femme sur 10** a indiqué avoir subi de la violence d'un partenaire intime au cours des cinq années précédentes. Cela représentait 126 500 Québécoises[4,5].
- Parmi les femmes qui consultent pour des services de santé reproductive (rendez-vous gynécologique, interruption de grossesse (IG), contraception, etc.), environ 40 % auraient déjà vécu de la violence d'un partenaire intime[6]. Les adolescentes et les jeunes femmes rapportent les taux plus élevés[7].

Ainsi, plusieurs des femmes que vous rencontrez ont vécu ou sont à risque de vivre de la violence entre partenaires intimes.

Certains gestes de violence entre partenaires intimes menacent directement l'autonomie ainsi que la santé sexuelle et reproductive des femmes et des personnes susceptibles d'être enceintes. C'est ce qu'on identifie comme étant de la coercition reproductive.

Lorsque les femmes vivent de la coercition reproductive, elles s'inquiètent notamment d'avoir contracté une ITSS ou d'avoir une grossesse non désirée. Elles vont alors vers des services de santé pour obtenir un dépistage d'ITSS, une consultation pour la prescription de contraceptifs ou d'une contraception orale d'urgence, ou encore un rendez-vous pour une interruption de grossesse (IG). Elles peuvent aussi avoir besoin de parler dans un espace sécuritaire et d'être écoutées sans jugement.

Vous êtes l'une des personnes susceptibles de recevoir ces femmes lors de vos activités professionnelles. En raison de votre rôle, vous pouvez identifier la présence de coercition reproductive, qu'elle s'inscrive dans un contexte de violence entre partenaires intimes, qu'elle soit l'issue d'une agression sexuelle ou qu'elle ait lieu dans un contexte amoureux exempt d'autres formes de violence. Vous pouvez offrir un soutien à ces femmes, les valider et les diriger vers les ressources appropriées, le cas échéant. De plus, vous pouvez faire de l'éducation préventive au sujet de ces problématiques auprès des personnes que vous rencontrez.

« Poser des questions sur la violence entre partenaires intimes prend trop de temps et risque d'offusquer la femme. »

La violence entre partenaires intimes est une problématique complexe. Plusieurs professionnel·le·s de la santé et intervenant·e·s sont réticent·e·s à aborder la violence puisque son identification prend du temps. D'autres sont mal à l'aise ou craignent d'offusquer les femmes en posant ces questions. Ces craintes sont communes et légitimes, mais ne doivent pas être un frein à l'intervention.

Ce guide offre un soutien dans l'intervention. Il propose des étapes courtes et ciblées (voir la section *Exemples d'interventions brèves*), qui ne représentent pas une surcharge de travail.

Poser des questions sur la violence entre partenaires intimes peut vous rendre mal à l'aise. Ce guide propose des exemples de formulation pour faciliter l'intervention.

Les études démontrent que les femmes ne sont pas offusquées de se faire poser des questions sur la violence entre partenaires intimes[8,9]. Cela peut les inciter à se questionner sur leur relation. Elles sont aussi plus enclines, par la suite, à aller chercher du soutien ou à quitter la relation[10].

Maintenant que nous avons établi que vous êtes une personne-ressource, pourquoi s'informer sur ce sujet ?

Aux États-Unis, une intervention brève portant spécifiquement sur la coercition reproductive a été mise sur pied dans certains services de santé[11]. Cette stratégie d'intervention a été évaluée pour déterminer son efficacité dans la réduction de la violence entre partenaires intimes et de la coercition reproductive.

Cette intervention a des visées multiples **:**

- Elle poursuit un **objectif d'éducation** auprès des femmes pour les informer de ce que sont la violence entre partenaires intimes et la coercition reproductive. Comme plusieurs femmes n'identifient pas la violence d'emblée, la description de ce qui constitue de la violence peut les aider à reconnaître de telles situations. Cet objectif d'éducation permet également aux femmes ne vivant pas de violence de se renseigner sur les comportements coercitifs et les distinctions entre une relation intime égalitaire et une relation marquée par le contrôle et la violence.

- L'intervention intègre aussi la **réduction des méfaits** dans les situations de coercition reproductive, notamment en proposant aux femmes des méthodes contraceptives peu détectables par leur partenaire (p. ex. **:** dispositif intra-utérin, communément appelé stérilet) ou en facilitant l'accès à la contraception orale d'urgence. Bien sûr, les stratégies de réduction des méfaits ne sont pas suffisantes en soi lorsqu'il est question de violence faite aux femmes. Ces stratégies doivent être accompagnées de soutien psychosocial ou de référencement vers des organismes psychosociaux spécialisés en violence. Avec ce soutien, les femmes pourront évaluer la qualité de leur relation intime.

- Enfin, l'intervention inclut une forme **d'implication des professionnel·le·s de la santé et des services sociaux et des intervenant·e·s dans le référencement**. Cela signifie d'accompagner les femmes dans leur recherche de services (p. ex. encourager la femme à contacter une ressource en présence d'une intervenante, appeler l'organisme pour recommander la femme et établir un premier rendez-vous), plutôt que de simplement les y diriger.

Cette stratégie d'intervention simple, mais ciblée, a démontré son efficacité : elle augmente l'identification et la prise en charge à la fois des situations de coercition reproductive et de violence entre partenaires intimes, en plus de prévenir ces situations auprès des femmes en général.

Comment utiliser ce document ?

Dans le présent document, nous utilisons les termes « fille », « femme » et « santé des femmes ». Cependant, il est important de reconnaître que les services de santé sexuelle et reproductive « pour femmes » s'adressent en réalité à toute personne ayant besoin de ces services pour maintenir une santé gynécologique et un bien-être reproductif. Certaines de ces personnes ne s'identifient pas en tant que femme. Il peut s'agir de personnes dont l'identité est non genrée (p. ex. : queer, non-binaire, agenre, genderfluid) ou trans (p. ex. : homme trans). Les services gynécologiques et obstétriques doivent donc être appropriés, inclusifs et adaptés aux besoins de toutes les personnes concernées.

Objectifs du guide

- Informer les professionnel·le·s de la santé et des services sociaux et les intervenant·e·s qui œuvrent auprès de femmes susceptibles de devenir enceintes au sujet de la coercition reproductive et de la violence entre partenaires intimes.
- Sensibiliser les professionnel·le·s et les intervenant·e·s aux meilleures pratiques d'intervention sur ces enjeux.
- Partager, avec les professionnel·le·s et les intervenant·e·s, les ressources disponibles et les formations sur la violence entre partenaires intimes et sur la coercition reproductive.

Descriptif du contenu

Ce document vise à détailler ce qu'est la coercition reproductive et la violence entre partenaires intimes. Vous y trouverez **:**

- la définition de la coercition reproductive et ses manifestations ;
- les répercussions associées à cette problématique ;
- les contextes pouvant augmenter la vulnérabilité ;
- des pistes pour bonifier les pratiques d'intervention.

Plus spécifiquement, vous y trouverez les points saillants de l'approche de soins sensibles aux traumas et de la sécurisation culturelle, de même que les stratégies d'intervention pour l'identification de la coercition reproductive. Un outil créé à l'intention des femmes sera aussi détaillé afin que vous puissiez le leur remettre et répondre à leurs questions, le cas échéant. De plus, vous trouverez des ressources spécialisées vers lesquelles vous pourrez diriger les femmes, au besoin, ainsi que des ressources complémentaires pour vous.

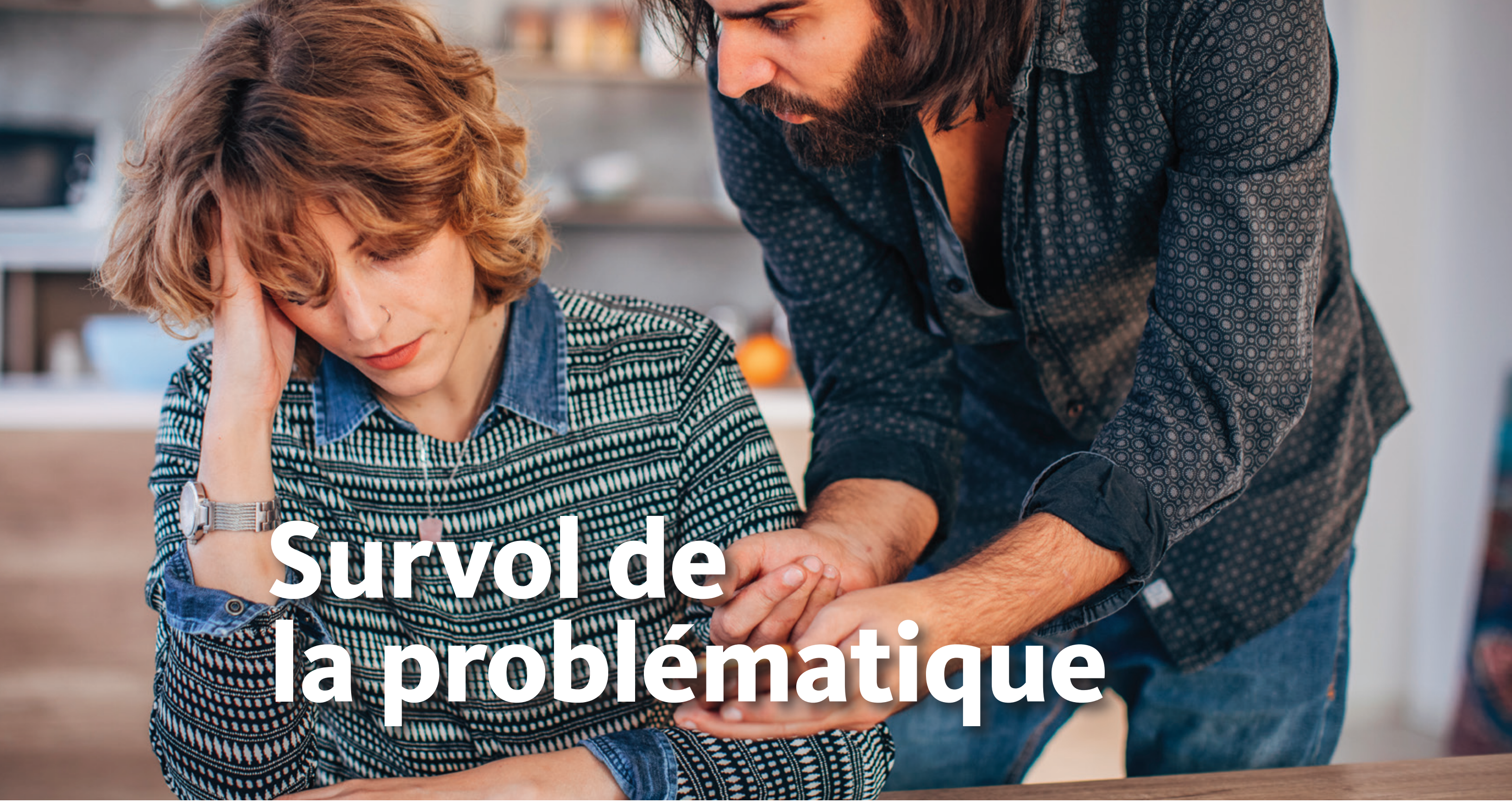

Survol de la problématique

Qu'est-ce que la coercition reproductive ?

La coercition reproductive renvoie à des comportements qui :

- interfèrent avec la contraception et la planification des naissances ;
- réduisent l'autonomie reproductive ;
- limitent la sélection de moyens contraceptifs, leur négociation et leur usage.

La coercition reproductive s'inscrit dans un désir de pouvoir et de contrôle sur l'autre personne. Elle peut se manifester dans une relation où est déjà présente une dynamique de violence conjugale. La littérature démontre des liens entre la coercition reproductive et la violence entre partenaires intimes. Une étude illustre que les adolescentes vivant de la violence entre partenaires intimes rapportaient aussi de la pression verbale de la part de leur partenaire pour devenir enceintes ou encore de l'interférence avec la contraception qu'elles utilisent. Ces comportements ont mené, dans certains cas, à des grossesses non désirées[12]. Ainsi, la coercition reproductive s'additionne aux autres comportements de violence vécus dans le contexte intime.

La coercition reproductive peut aussi se manifester au sein d'une relation intime ou amoureuse sans qu'il y ait présence d'autres comportements de violence.

Quelles formes peut prendre la coercition reproductive ?

Les études tendent à indiquer qu'il ne s'agit pas d'un phénomène isolé : entre 8 % et 27 % des femmes en âge d'être enceintes aux États-Unis rapportent avoir subi de la coercition reproductive[13-15]. Plusieurs formes de coercition reproductive sont actuellement documentées dans les études scientifiques.

SABOTAGE CONTRACEPTIF

Cette forme de coercition reproductive implique l'interférence avec la méthode contraceptive employée par la femme dans le but de provoquer une grossesse[10]. Une ou plusieurs tactiques peuvent être utilisées : cacher, saboter ou détruire les pilules contraceptives ; briser ou percer les condoms ; ne pas utiliser le retrait comme convenu ; retirer l'anneau vaginal, les timbres contraceptifs ou le stérilet ; mentir sur une vasectomie ; empêcher l'achat de contraceptifs ou refuser de contribuer financièrement à leur achat dans le but de réduire leur accès ou leur usage.

PRESSIONS LIÉES À LA GROSSESSE

Ces pressions visent la promotion de la grossesse, sans égard aux intentions reproductives de la femme[14,16,17]. Cela peut se traduire par des menaces, du contrôle, du chantage, du harcèlement ou encore l'imposition de rapports sexuels non protégés dans le but de provoquer une grossesse.

COERCITION LORS DE LA GROSSESSE

Il s'agit de comportements coercitifs et de violence employés afin d'imposer l'issue de la grossesse[16]. Cela peut impliquer de blesser physiquement la partenaire de façon à provoquer une fausse couche ou d'empêcher l'accès à l'avortement par différents moyens (p. ex. : en contrôlant ses sorties, en empêchant l'accès à la voiture, etc.), de sorte que la grossesse soit menée à terme[18]. Il pourrait aussi s'agir de forcer une personne à interrompre une grossesse alors qu'elle souhaite la poursuivre.

Saviez-vous que...

Le sabotage contraceptif a aussi fait l'objet d'un jugement de la Cour suprême du Canada en 2014. Dans ce jugement, l'auteur des gestes a été accusé d'agression sexuelle grave. Comme le consentement à l'activité sexuelle impliquait l'usage d'un condom intact, son absence rend le consentement invalide. Pour lire le jugement complet, référez-vous au site Web de la Cour suprême du Canada et consultez le cas ***R.C. Hutchinson, 2014 CSC 19 C.F.R***.

Selon nos travaux passés et de nouvelles études, il est possible d'identifier d'autres gestes relevant de la coercition reproductive. En voici quelques-uns :

- **Interférence contraceptive**[24] : renvoie au retrait non consensuel du condom lors d'une relation sexuelle (nommé *stealthing* en anglais). Contrairement au sabotage contraceptif, l'interférence contraceptive dans une relation sans engagement amoureux ne vise pas à provoquer délibérément une grossesse. Il est plutôt question de contrôler les paramètres de la relation sexuelle, par exemple qu'elle se déroule sans condom, ce qui affecte la capacité de se protéger contre des grossesses non désirées ou des ITSS. Le partenaire sexuel priorise son plaisir sexuel, au détriment de la santé sexuelle et reproductive de sa partenaire.

« *C'est la situation où la personne enlève le condom sans consentement et puis... hum... sans demander la permission ni rien. Puis, continuer comme si de rien n'était alors que... t'sais... on a accepté la relation en sachant qu'on allait porter le condom et pas... pas sans condom, là !* (Maria, 18 ans)[20] »

- **Contrôle contraceptif**[20] **:** se traduit par un suivi accru et envahissant de l'usage de contraceptifs de la femme. Cela peut se manifester de diverses façons : s'assurer de voir la prise de la pilule contraceptive, exiger de suivre le cycle menstruel, s'assurer de la survenue des menstruations, imposer la prise de contraception d'urgence si un risque est perçu, etc. Le contrôle contraceptif inclut aussi d'imposer une méthode contraceptive jugée plus fiable pour éviter la survenue d'une grossesse, sans égard aux préférences contraceptives de la femme.

« *Donc, les manifestations les plus fréquentes, c'était de me donner ma pilule pour que je la prenne (devant lui), de me demander si j'étais dans ma semaine, puis d'aller acheter les pilules avec moi. Ça, c'est vraiment ce que j'ai vécu le plus souvent. Dernièrement, c'était de me parler vraiment souvent du stérilet en cuivre... vraiment, vraiment souvent.* (Lilianne, 24 ans)[20] »

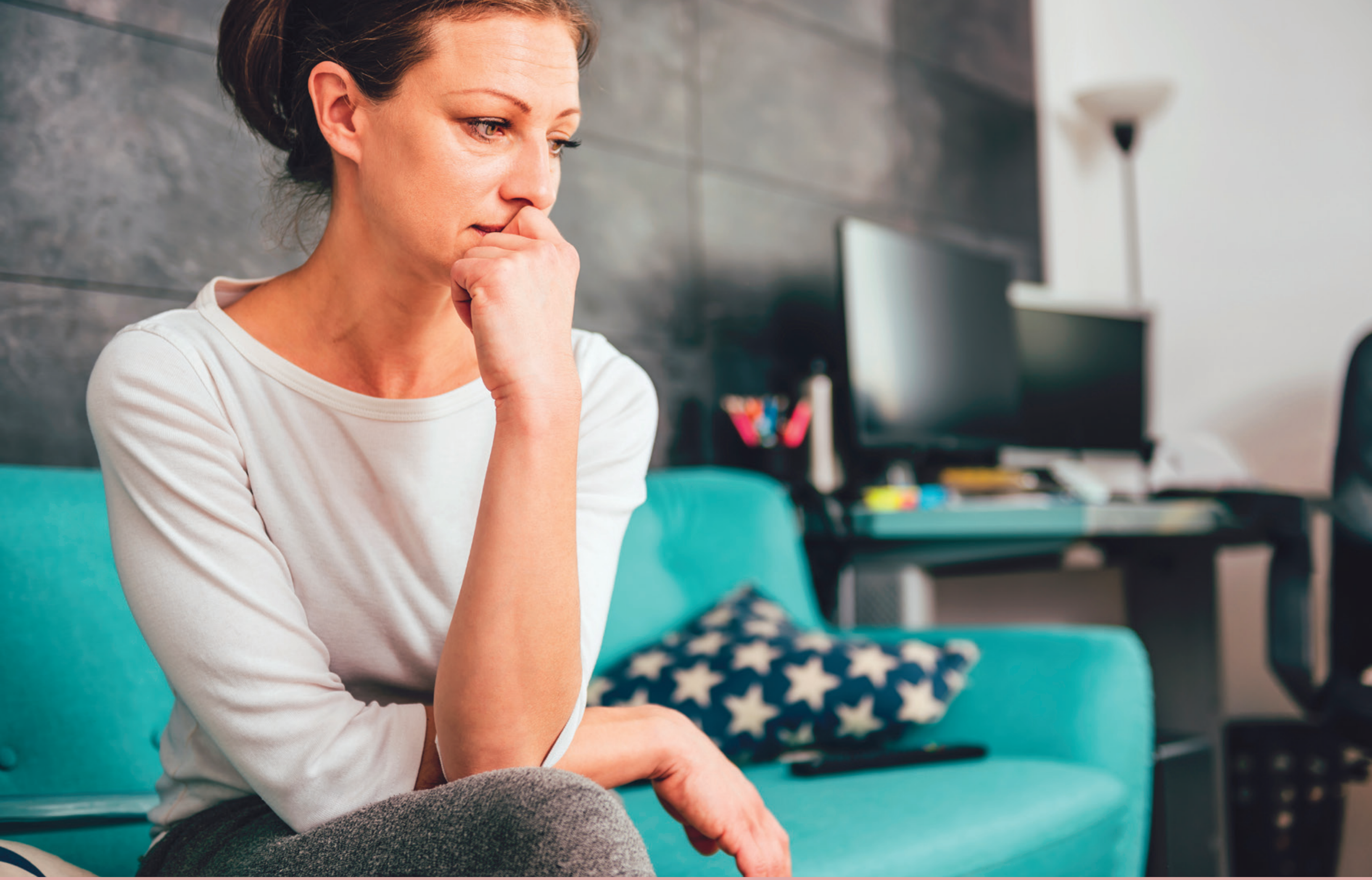

Les manifestations de coercition reproductive peuvent différer selon le contexte relationnel[19] :

- Lorsqu'elle se présente au sein d'une relation conjugale, elle vise généralement à imposer une grossesse ou, au contraire, à imposer l'arrêt de la grossesse.
- Lorsqu'elle se présente au sein d'une relation intime où il n'y a pas d'engagement amoureux (p. ex. : partenaire d'un soir, fréquentation), elle vise principalement à entraver le port du condom (nommé « interférence contraceptive »).

Des gestes de coercition reproductive peuvent aussi être posés par des personnes avec qui il n'y a pas de liens intimes ou amoureux, comme un membre de la famille ou de la belle-famille, ou encore une personne en situation d'autorité. Les gestes posés visent à limiter l'autonomie reproductive et ciblent principalement l'imposition d'une grossesse ou son arrêt. Ces gestes visent à contrôler la contraception ou la planification des naissances. Par exemple, il peut s'agir d'infliger la stérilisation forcée aux femmes autochtones[21] ou encore de limiter les choix reproductifs des femmes racisées[22,23].

Les vignettes présentées à la page suivante présentent deux formes de coercition reproductive, soit l'interférence contraceptive et les pressions liées à la grossesse. Elles sont inspirées des témoignages partagés par des femmes rencontrées lors d'un projet de recherche sur la coercition reproductive[20].

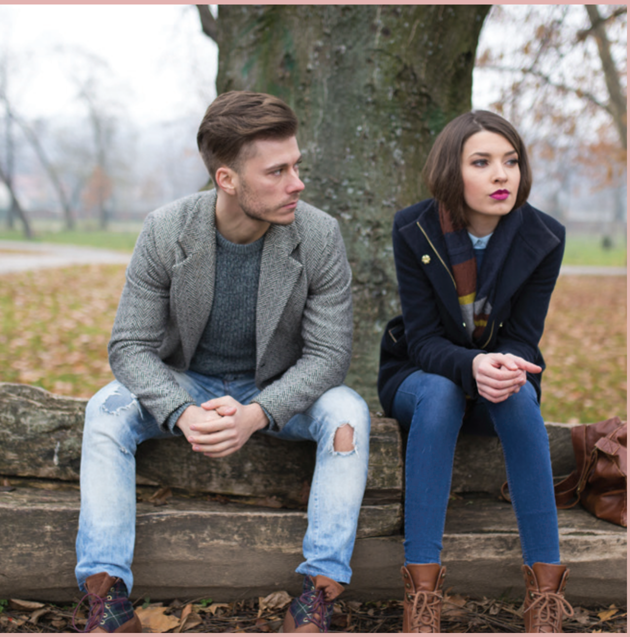

Sophie fréquente depuis peu Matthias, un jeune homme, qui assiste au même cours d'université qu'elle. Ils se sont rencontrés quelques fois en dehors du cours. Ils ont eu des relations sexuelles, qui ont toujours été agréables. Julie doit négocier le port du condom avec Matthias à chaque relation sexuelle, mais elle considère que c'est une situation assez banale, bien que cette négociation soit lourde à assumer. Lors de leur dernière relation sexuelle, Julie a donné un condom à Matthias pour qu'il le mette. Elle s'est rendu compte, à la fin de la relation, qu'il ne portait pas le condom, après avoir feint de le mettre. Il s'est retiré avant d'éjaculer.

Sophie a alors été prise de panique. Elle lui a dit qu'elle n'utilisait pas d'autre moyen de contraception et qu'elle s'inquiétait à l'idée qu'il lui avait transmis une ITSS. Matthias lui a répondu qu'il n'y avait aucun risque, puisqu'il s'était retiré. Il lui a aussi dit qu'elle ne devait pas s'en faire avec les ITSS puisqu'il ne fréquentait pas une autre femme en même temps qu'elle.

Julie-Kim est en couple avec son partenaire, Tam, depuis près de trois ans. Tous deux approchent la trentaine et leurs familles respectives ne se gênent pas pour leur demander à quel moment ils comptent avoir un enfant. Julie-Kim ne sait pas encore si elle souhaite avoir des enfants, mais son partenaire, lui, semble en vouloir maintenant. En fait, il lui fait des commentaires presque tous les jours concernant son désir d'avoir un enfant : *« As-tu vu son bébé ? J'ai tellement hâte d'avoir le nôtre ! »* ; *« Je veux vraiment un enfant avant d'avoir trente ans »* ; *« Arrête ta pilule, je veux qu'on essaie maintenant »*. Plus le temps avance, plus les remarques sont fréquentes, insistantes et parfois méchantes. Il a commencé à lui dire qu'elle ne l'aimait pas vraiment parce qu'autrement, elle aurait un enfant avec lui. Il a aussi menacé de la quitter si elle ne devenait pas enceinte prochainement. Dernièrement, il l'a insultée en lui disant qu'elle ne serait probablement pas une bonne mère, puisqu'elle continue de sortir prendre un verre avec ses ami·e·s, ce qui démontre, selon lui, qu'elle n'est qu'une « égoïste sans-cœur ».

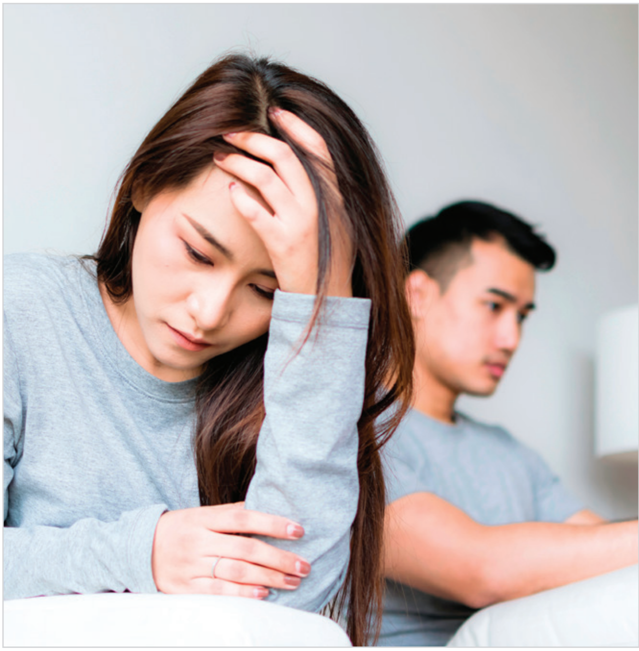

Quelles sont les répercussions de la coercition reproductive ?

Sans être exhaustive, la liste ci-dessous présente quelques-unes des principales répercussions de la coercition reproductive sur la santé physique, psychologique, sexuelle et reproductive des personnes qui en sont victimes. Certaines répercussions s'apparentent à celles qui sont rapportées dans les cas de violence sexuelle ou de violence conjugale.

RÉPERCUSSIONS SUR LA SANTÉ PHYSIQUE ET PSYCHOLOGIQUE

- Stress et anxiété
- Isolement social
- Honte
- État de choc, confusion
- Sentiment de perte de contrôle
- Baisse de la confiance en soi
- Trouble du sommeil ou cauchemars récurrents
- Idéations suicidaires
- Colère

RÉPERCUSSIONS SUR LA SANTÉ SEXUELLE ET REPRODUCTIVE

- Grossesse non désirée
- Avortement
- Perte du désir d'avoir un enfant
- Baisse du désir sexuel et du plaisir sexuel
- Infections transmissibles sexuellement

RÉPERCUSSIONS SUR LE BIEN-ÊTRE RELATIONNEL

- Diminution de l'intérêt envers les relations amoureuses et intimes
- Méfiance par rapport aux hommes

Les extraits suivants présentent les répercussions que peut avoir la coercition reproductive sur une personne. **Le premier extrait illustre le sentiment de panique et de confusion** qui peut être ressenti après avoir subi un retrait non consensuel du condom avec un partenaire sexuel.

> « *Sur le coup, je suis sous le choc, je panique. Je me suis réveillée le lendemain matin, je ne me sentais vraiment pas bien, j'étais tellement confuse... J'étais juste vraiment désorientée. Je ne comprenais plus ce qui se passait. J'avais plein d'émotions contradictoires.* (Nadine, 26 ans)[19] »

Dans le cadre des relations qui présentent d'autres comportements de violence s'ajoute un sentiment de crainte face au partenaire et au contrôle qu'il exerce. **L'extrait suivant illustre l'anxiété et la peur ressenties** par une femme ayant subi de la coercition lors de la grossesse dans un contexte de violence entre partenaires intimes.

> « *Il y a plusieurs fois où j'ai fait des tests de grossesse, où j'étais vraiment stressée. Je les ai faits en cachette. Je n'avais pas envie qu'il soit là ou même qu'il les trouve. Je suis allée les jeter dans le parc pour ne pas qu'il les trouve [...]. Je ne voulais pas qu'il sache que je suis enceinte. Parce que ça me laissait plus de... ça me laissait une plus grande liberté de choisir après, si jamais j'étais enceinte.* (Rebecca, 29 ans)[19] »

Certaines personnes vivront des conséquences à court terme, alors que d'autres verront ces conséquences se manifester plus tard. Plusieurs conséquences peuvent aussi se combiner. Que les répercussions soient clairement identifiées ou non par la personne victime au moment où vous la rencontrez, sa santé et son bien-être peuvent être affectés [19,24-27].

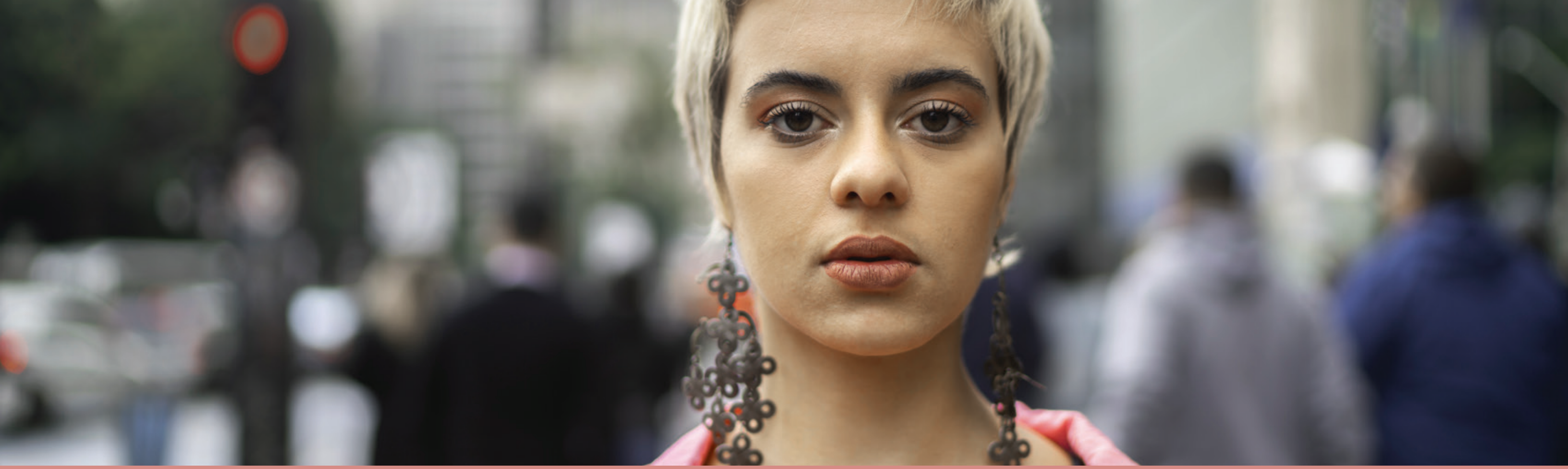

Quels contextes peuvent augmenter la vulnérabilité ?

Pour l'instant, les études qui documentent les facteurs de risque liés à la coercition reproductive sont limitées. Il est possible de croire que les contextes de vulnérabilité liés à la violence entre partenaires intimes, qui sont bien documentés, soient similaires à ceux liés à la coercition reproductive.

Dans le cadre de groupes de discussion sur ce sujet, plusieurs professionnel·le·s de la santé et intervenant·e·s nous ont rapporté les contextes observés sur le terrain pouvant augmenter la vulnérabilité :

Faible niveau de littératie en santé : avoir des compétences limitées pour trouver, comprendre, évaluer et utiliser l'information en santé a des effets sur la santé reproductive. Une faible littératie en santé pourrait ainsi être liée à une moindre propension à recourir aux services. Il peut aussi s'agir de s'appuyer sur des informations limitées ne permettant pas une prise de décision éclairée[28,29].

Rapport de pouvoir inégal avec le partenaire intime : être jeune est lié à une augmentation du risque de vivre de la coercition reproductive (p. ex. : être une adolescente ou une adulte de moins de 30 ans)[30,31]. De plus, les femmes ayant vécu du sabotage contraceptif ont tendance à fréquenter des partenaires plus âgés qu'elles, comparativement à celles qui n'en rapportent pas[32].

Trajectoire de migration : immigrer dans un nouveau pays peut générer un stress important et être accompagné de conditions de vie précaires, notamment une diminution du soutien social, une méconnaissance des lois et une situation socioéconomique difficile[33,34]. Le coût des services de santé, incluant la santé reproductive pour une personne qui n'est pas admissible à la couverture provinciale, est prohibitif[35].

Barrières linguistiques : ne parler ni français ni anglais est sans contredit un obstacle à l'accès aux services de santé, aux informations médicales disponibles en ligne, à l'identification d'un·e professionnel·le de la santé ou des services sociaux, à la prise de rendez-vous médical, à la divulgation de la situation vécue, etc.[36]

Indicateurs à surveiller lors d'une consultation

La violence entre partenaires intimes et la coercition reproductive sont des problématiques qui sont généralement cachées ou minimisées par les femmes qui les vivent. Ainsi, il peut être ardu pour un·e professionnel·le de la santé ou un·e intervenant·e de déceler leur présence.

Voici quelques exemples qui peuvent mettre la puce à l'oreille :

- La femme a le regard fuyant, semble nerveuse et a une apparence négligée. Elle pleure beaucoup et semble hésitante.
- L'homme impose sa présence lors du rendez-vous médical de sa partenaire. Il répond aux questions à sa place et lui laisse peu de place lors de la consultation.
- La personne choisit de ne pas donner son numéro de téléphone ou son adresse à la réception ou lors de la consultation.
- La femme dit que son partenaire n'est pas au courant et qu'il ne doit pas être informé du rendez-vous médical ou psychosocial.
- La femme demande à ce que la méthode de contraception soit peu détectable (p. ex. : pose de stérilet).
- La femme a eu recours, à plusieurs reprises au cours des derniers mois, à un dépistage d'ITSS, à une IG ou à une prise de contraceptifs oraux d'urgence.
- Lors de la rencontre, le téléphone de la femme sonne sans cesse.

Bien sûr, la présence d'un ou de plusieurs de ces indicateurs ne signifie pas systématiquement qu'il y ait présence de coercition reproductive ou de violence entre partenaires intimes. Toutefois, selon les professionnel·le·s de la santé ou des intervenant·e·s que nous avons rencontré·e·s, cela permet d'ouvrir la discussion sur le sujet.

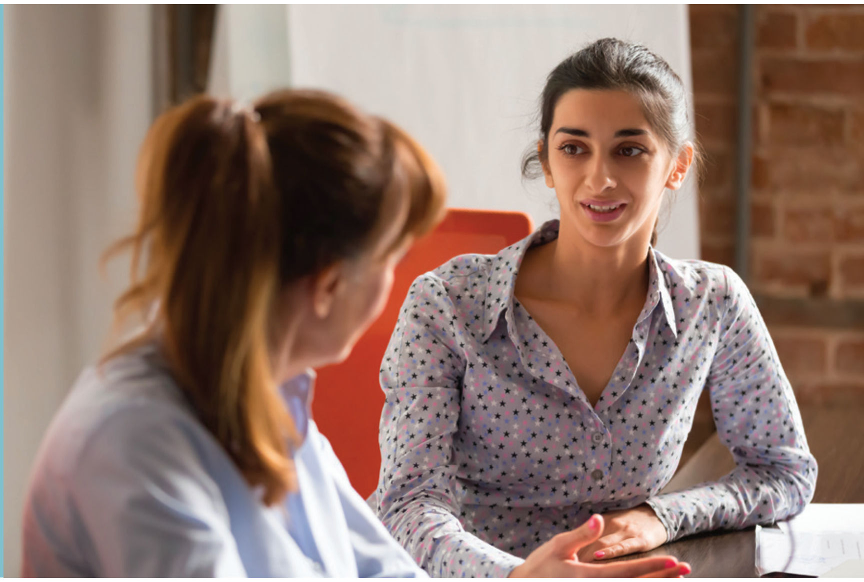

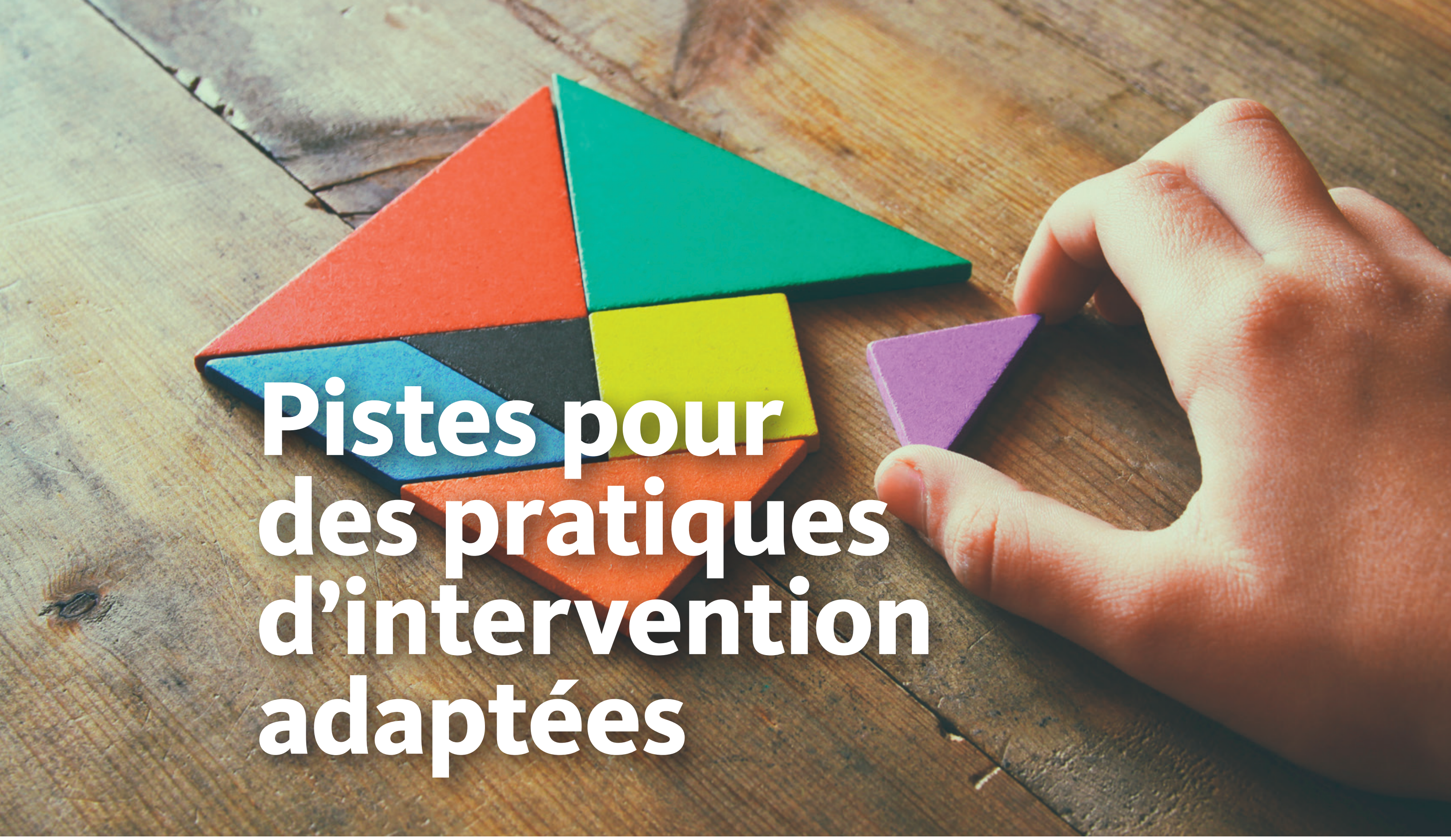

Pistes pour des pratiques d'intervention adaptées

Différentes approches ont été développées pour favoriser des interventions respectueuses et sécurisantes pour les personnes concernées. Nous vous présentons deux approches que nous estimons pertinentes pour les soins de santé reproductive et la violence, l'approche de soins sensibles aux traumas et la sécurisation culturelle.

Approche de soins sensibles aux traumas

L'approche de soins sensibles aux traumas vise à comprendre l'impact des expériences traumatisantes dans la vie des personnes victimes, sans avoir à questionner ou à traiter directement ces traumatismes.

Cela signifie d'adopter un savoir-être visant l'intégration d'interactions bienveillantes et inclusives des diverses réalités des personnes consultant pour des services[37].

Il s'agit de s'intéresser aux expériences potentiellement traumatisantes que peuvent avoir vécues les femmes, de les prendre en considération avec respect et bienveillance, sans toutefois exiger ou forcer leur dévoilement[38,50].

QUI DEVRAIT ÊTRE FORMÉ POUR APPLIQUER CETTE APPROCHE ?

Toutes les personnes travaillant dans l'organisation ou l'organisme : les intervenant·e·s psychosociaux·ales, les personnes qui travaillent à l'accueil ou à la prise de rendez-vous, les professionnel·le·s de la santé, etc. Comme ces dernières entrent en contact avec des personnes pouvant avoir vécu des expériences traumatisantes, cette approche devrait s'intégrer dans la culture organisationnelle. Cela implique aussi que le personnel sache adopter des procédures respectueuses (p. ex. : demander le consentement avant le début d'un examen gynécologique) ou empathiques (p. ex. : reconnaître ce qui a été vécu, reconnaître la douleur de la personne)[39].

L'approche de soins sensibles aux traumas demande une certaine introspection, préalable à sa mise en œuvre. Comme elle s'appuie fortement sur le savoir-être, l'ensemble du personnel est invité à prendre conscience de ses valeurs, de ses pratiques et de ses perceptions à l'égard de la violence, des traumas et des personnes victimes. Il s'agit d'une démarche de réflexion en continu.

MESSAGES CLÉS DE L'APPROCHE DE SOINS SENSIBLES AUX TRAUMAS

- Être conscient·e que la personne qui consulte ait pu vivre une expérience traumatisante ;
- Reconnaître le besoin de sécurité physique et émotionnelle de la personne qui consulte et interagir de manière à ce qu'elle se sente en sécurité avec vous ;
- Inclure la personne dans son processus de guérison en favorisant la collaboration (p. ex. : présenter les options qui s'offrent à elle, incluant l'option de ne pas s'engager dans des services proposés) ;
- Croire à la force et à la résilience de la personne qui consulte ;
- Garantir des stratégies d'intervention sensibles à l'identité personnelle et sociale de la personne qui consulte (en référence aux groupes qui sont opprimés en raison, entre autres, de leur appartenance ethnoculturelle, de leur genre ou de leur orientation sexuelle). Faire preuve de sensibilité face à la marginalisation du groupe auquel la personne appartient augmente l'efficacité de l'approche.

Cette approche se prête bien au soutien clinique pouvant être offert aux femmes ayant vécu de la coercition reproductive, dans un contexte de violence entre partenaires intimes ou non[40]. Pour plus d'informations sur l'approche, consultez la section *Pour en savoir plus*.

La sécurisation culturelle

La sécurisation culturelle a été développée dans les années 1980 par des professionnelles de la santé autochtone, en Nouvelle-Zélande, afin d'offrir des services culturellement adaptés aux personnes Maori consultant le système de santé[49]. Cette approche est maintenant intégrée dans différents secteurs et vise l'adoption de pratiques cliniques et d'intervention respectueuses de toutes personnes consultant des services de santé ou sociaux. Il existe plusieurs stratégies et postures permettant d'adopter des pratiques d'intervention inclusives auprès d'une personne qui n'a pas le même bagage culturel que soi. Cela inclut l'identification de différents rapports de pouvoir inégaux, en reconnaissant que la discrimination institutionnelle ou systémique et les effets du colonialisme ont un impact sur l'accès et la qualité de soutien dans les services de santé[41,42].

Cette approche reconnaît qu'il existe des groupes de personnes qui sont opprimés en raison de leur appartenance culturelle. Cette dernière ne se limite pas à la culture au sens unique de l'origine ethnique. Elle renvoie aussi au partage de croyances, d'idées et de significations partagées, de valeurs, de façons d'être et d'expressions langagières[41,43].

Le but de la sécurisation culturelle est de contribuer à la réduction des inégalités dans l'accès à la santé, non pas en traitant différemment les personnes ayant une appartenance ethnoculturelle minoritaire, mais en reconnaissant les rapports de pouvoir inégaux qui sont présents[41,44]. Elle s'apparente aux pratiques antioppressives[45,46] et vise une adaptation des interventions en fonction de ces enjeux.

La sécurisation culturelle demande de prendre conscience de sa propre culture et de réfléchir à son impact dans les interventions faites auprès des personnes qui consultent. Cela signifie, par exemple, de tenir compte des valeurs, des croyances et des normes qui guident nos comportements. Il est commun de penser que lorsque nous appartenons au groupe dominant (p. ex. : être un·e Québécois·e à la peau blanche francophone au Québec), nous sommes culturellement « neutre »[41]. Or, ce n'est pas le cas. Faire cet exercice permet de prendre conscience de son bagage culturel personnel, qui a inévitablement une influence dans une relation de services de santé ou de soutien psychosocial.

MESSAGES CLÉS DE LA SÉCURISATION CULTURELLE[47-49]

- Sonder les besoins et les attentes de la personne qui consulte, sans présumer les connaître d'avance ;
- Travailler avec cette personne afin de trouver des options adaptées à sa situation ;
- Être conscient·e des enjeux de pouvoir dans la relation entre le·la professionnel·le ou l'intervenant·e et la personne qui consulte.

La sécurisation culturelle est ressentie par une personne appartenant à un groupe minoritaire ou opprimé lorsqu'elle estime que les prestataires des services communiquent avec elle de façon respectueuse et inclusive, tout en encourageant et en valorisant sa prise de décision[44]. Pour plus d'informations sur l'approche, consultez la section *Pour en savoir plus*.

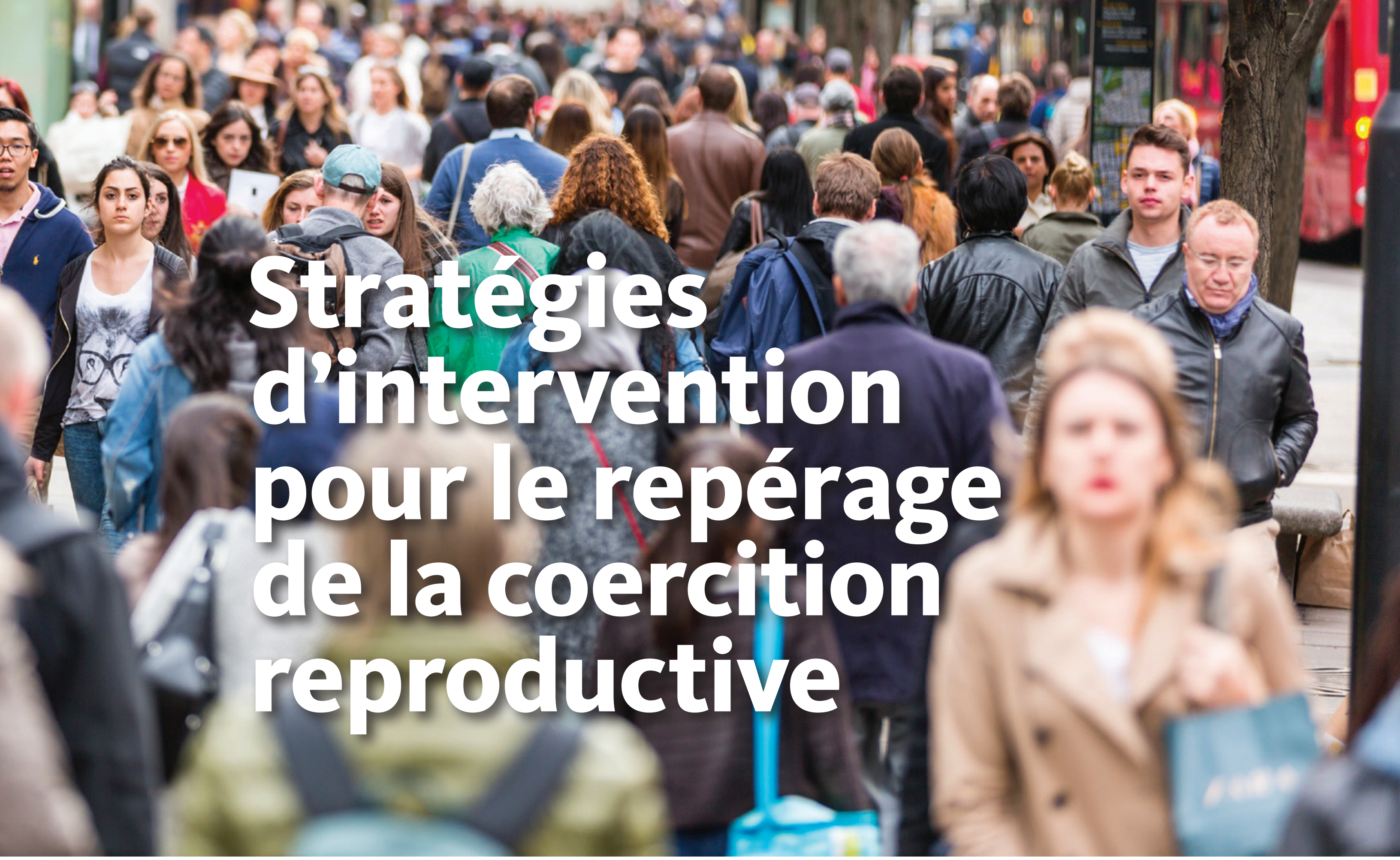

Stratégies d'intervention pour le repérage de la coercition reproductive

Comme professionnel·le de la santé ou intervenant·e psychosocial·e, vous pouvez contribuer à réduire les conséquences négatives vécues et la prévalence de la coercition reproductive. En adoptant une approche de soins sensibles aux traumas, en intégrant la sécurisation culturelle à votre pratique et en étant renseigné·e sur la problématique, vous pouvez faire une différence sur les plans de la santé et du bien-être global des filles et des femmes.

Cette section propose quatre grandes étapes d'intervention qui pourront vous guider et vous outiller sur la manière d'intégrer le concept de coercition reproductive dans votre travail auprès des femmes. Ces quatre étapes sont les suivantes :

**ACCUEILLIR**	**IDENTIFIER**
**SOUTENIR**	**DIRIGER**

Vous verrez qu'une intervention minimale sur la coercition reproductive ne demande pas beaucoup de temps et ne requiert pas une formation particulière.

ACCUEILLIR

Avant toute intervention, assurez-vous de proposer un endroit sécuritaire et confidentiel aux femmes qui vous consultent.

Certaines femmes allophones auront besoin de services d'interprètes lors de leur rendez-vous. Sans ces interprètes, elles auront de la difficulté à bien s'exprimer et à comprendre l'information qui leur est transmise et, par conséquent, à donner un consentement éclairé aux services qui leur sont offerts. La présence de services d'interprètes est donc importante. Dans le cas où aucun interprète n'est disponible, certaines organisations proposent à un partenaire ou à un membre de la famille de remplir ce rôle.

Il est préférable que les femmes ne soient pas accompagnées par une autre personne (c.-à-d. partenaire, membre de la famille, ami·e) au moment de la rencontre. Certaines femmes préféreront tout de même être accompagnées. Il faut souligner que cette situation peut compromettre le dévoilement de la violence vécue et menacer la sécurité d'une femme victime de violence. Le personnel soignant pourrait choisir de débuter la rencontre seulement avec la femme pour sonder son choix d'être accompagnée, ou pas, lors de la consultation.

À cette étape, une attention particulière portée aux comportements et à l'attitude de la femme pourra vous fournir des indicateurs quant à la présence possible de violence entre partenaires intimes ou de coercition reproductive (vous référez à la section *Les indicateurs à surveiller lors d'une consultation*).

CONFIDENTIALITÉ

Établissez au début de la rencontre les limites de la confidentialité.

Pour connaître les limites de la confidentialité propres à votre profession, vous pouvez consulter la ***Loi sur les services de santé et les services sociaux***, RLRQ c S-4.2, art. 19.0.1 et votre code de déontologie.

Voici un scénario visant à informer la personne qui vous consulte des limites de la confidentialité :

> *« Je suis ravi·e que vous soyez présente aujourd'hui pour notre rencontre de ......* [préciser le type de suivi]*. Avant qu'on débute, j'aimerais que vous sachiez que tout ce que vous allez me partager est confidentiel, sauf de très rares exceptions prescrites par la loi. Êtes-vous d'accord pour qu'on commence la rencontre ? »*

IDENTIFIER

Après avoir accueilli la personne, vous pouvez lui poser des questions sur la qualité de ses relations intimes. Ces différentes questions permettent d'avoir une meilleure idée de la situation dans laquelle elle se trouve.

Il vous est aussi possible de déterminer s'il y a des indicateurs de violence dans la relation intime et si la sécurité de la femme est menacée. Des questions précises sur la coercition reproductive vous permettront aussi d'identifier la présence de gestes visant à contrôler ou à nuire à sa santé et à son autonomie reproductive.

Dans cette section, vous retrouverez des recommandations d'interventions. Ainsi, il est suggéré d'adapter ces recommandations à votre réalité d'intervention, en vous appuyant sur votre expertise clinique.

Il est préférable d'amorcer la discussion en posant des questions sur le statut relationnel de la personne qui vous consulte. Selon son statut relationnel, vous pourrez explorer davantage la qualité de sa relation intime.

IDENTIFIER LA PRÉSENCE DE VIOLENCE ENTRE PARTENAIRES INTIMES

L'exemple de scénario présenté à la page suivante vous permet d'amorcer une discussion sur la situation relationnelle, puis sur la violence entre partenaires intimes. Le premier bloc de questions vous permettra d'explorer la qualité de la relation. En fonction des réponses reçus, vous pourrez poursuivre la discussion avec le deuxième bloc qui porte sur l'identification de la présence de violence.

Si, en répondant aux questions, la femme vous informe qu'elle est en relation avec une autre femme, il demeure pertinent de discuter de la qualité de sa relation pour savoir si elle vit des conflits ou de la violence dans son couple.

Vous pouvez aussi lui demander s'il lui arrive d'avoir des relations intimes avec des partenaires masculins. Si elle répond oui, vous pouvez poursuivre la discussion avec le reste des questions. Si elle répond non, vous pouvez tout de même lui remettre la brochure « ***Mon corps, mes choix… Est-ce bien le cas dans votre relation ?*** », pour lui permettre de lire les situations qui pourraient être vécues par des personnes de son entourage. Cette lecture contribuera à la sensibiliser à la problématique de la coercition reproductive.

« Vous êtes ici pour ..... [motif de la consultation]. *Avant d'en discuter davantage, j'aimerais d'abord vous demander :*

- *Êtes-vous en relation en ce moment avec quelqu'un ? Si oui, comment ça se passe dans votre relation avec votre partenaire ?*
- *Est-ce que vous vous sentez bien avec votre partenaire ?*
- *Vous arrive-t-il de vivre des conflits importants avec votre partenaire ? »*

Si la femme vous indique qu'elle vit des conflits importants ou qu'elle rapporte ne pas être bien dans sa relation, **poursuivez avec ces questions :**

Si la femme vous indique qu'elle n'a pas vécu ces situations, vous pouvez poursuivre avec la section **Identifier la présence de coercition reproductive.**

« Je suis désolée de savoir que vous vivez une situation difficile avec votre partenaire. Me permettez-vous de vous poser des questions plus personnelles sur votre relation ? J'aimerais m'assurer que vous êtes en sécurité :

- *Des femmes que je rencontre me disent qu'elles ont parfois peur de leur partenaire ou craignent ses réactions. Est-ce que c'est une situation que vous avez déjà vécue ou que vous vivez présentement ?*
- *Certaines me disent aussi que leur partenaire les a déjà blessées physiquement ou les a obligées à avoir une relation sexuelle contre leur gré. Est-ce que c'est quelque chose que vous avez déjà vécu ou que vous vivez présentement ? »*

Des réponses positives à ces questions vous indiquent que **la femme semble vivre de la violence entre partenaires intimes.**

Si la femme vous indique qu'elle n'a pas vécu ces situations, vous pouvez poursuivre avec la section **Identifier la présence de coercition reproductive.**

IDENTIFIER LA PRÉSENCE DE COERCITION REPRODUCTIVE

Bien que la brochure se concentre particulièrement sur la prévention des grossesses non désirées, ces discussions offrent l'opportunité d'aborder la prévention des ITSS. Ainsi, vous pouvez demander à la personne qui vous consulte comment se déroule l'usage de contraception visant à prévenir les ITSS lors de ses relations sexuelles (p. ex. : usage du condom, négociation de son usage). Il s'agit d'une bonne occasion pour la sensibiliser aux risques d'ITSS, en plus de proposer des moyens de protection qui pourraient s'avérer plus efficaces ou plus adaptés.

IDENTIFIER LA PRÉSENCE DE COERCITION REPRODUCTIVE

Après avoir posé des questions à la femme sur ses relations intimes, vous pouvez aborder le sujet de la coercition reproductive. De cette manière, vous pourrez recueillir des indicateurs plus spécifiques à la coercition reproductive : gestes posés par son partenaire visant à contrôler sa vie reproductive et sexuelle, désir de la femme d'obtenir un contraceptif peu détectable, inquiétudes de la femme à l'idée que son partenaire soit au courant de sa présence dans un établissement de santé et de services sociaux, etc.

Les questions portant sur la coercition reproductive vous permettront d'avoir une meilleure idée des besoins de la femme, du soutien approprié et des autres risques de santé et de sécurité potentiellement présents.

Les études démontrent que poser ces questions aux filles et aux femmes permet de les sensibiliser à cette problématique, ce qui réduit les risques qu'elles vivent de la coercition reproductive. Si elles vivent l'une de ces situations, elles seront aussi plus enclines à demander du soutien professionnel et à rompre avec le partenaire violent.

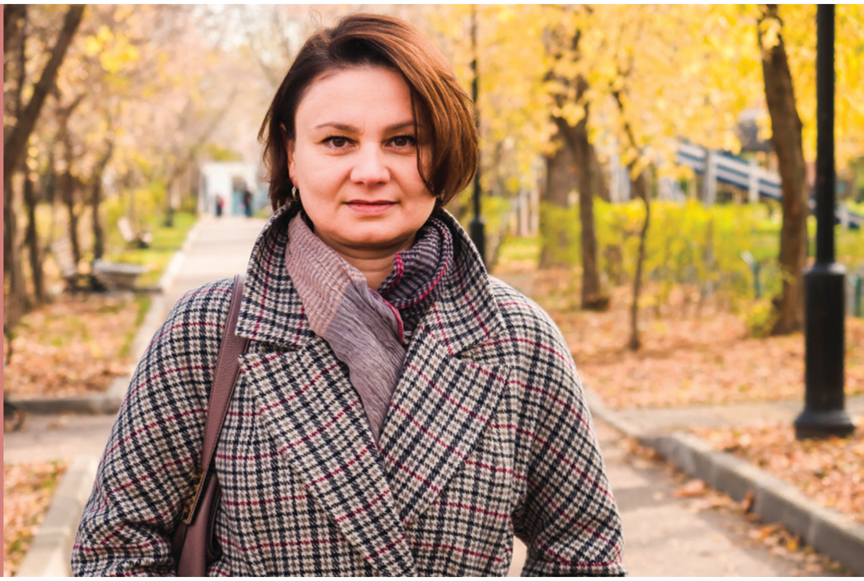

« Maintenant, si vous le voulez bien, on parlerait plus spécifiquement de votre santé sexuelle et reproductive. J'ai pris l'habitude de toujours poser ces questions aux femmes qui viennent me voir. Peut-être que c'est une situation que vous ne vivez pas personnellement, mais que quelqu'un de votre entourage vit. C'est la raison pour laquelle je préfère vous poser ces questions :

- *Utilisez-vous une méthode de contraception actuellement ?*
  - ↳ *Si oui, laquelle ? Est-ce votre choix personnel d'utiliser cette méthode de contraception en particulier ?*
  - ↳ *Si non, est-ce que c'est votre choix personnel de ne pas utiliser de contraception ? Souhaiteriez-vous utiliser une méthode de contraception ?*
- *Est-ce que l'utilisation de cette méthode de contraception cause parfois des conflits ou des tensions avec votre partenaire ?*
- *Est-ce qu'il vous arrive d'avoir peur de demander à votre partenaire de mettre un condom ?* [si applicable selon la méthode de contraception]
- *Est-ce que votre partenaire vous a déjà empêché d'utiliser une méthode de contraception (p. ex. : jeter vos pilules contraceptives, retirer le condom sans vous avertir, etc.) ? »*

Des réponses positives à ces questions vous indiquent que **la femme est susceptible de vivre de la coercition reproductive** sous la forme de sabotage contraceptif, d'interférence contraceptive ou de contrôle contraceptif.

Si la femme vous répond qu'elle n'a pas vécu ces situations, remettez-lui quand même la brochure « **Mon corps, mes choix... Est-ce bien le cas dans votre relation ?** ». Cela pourra la sensibiliser à la problématique de la coercition reproductive.

Si vous obtenez des réponses positives aux questions du bloc précédent, vous pouvez poursuivre avec celles-ci.

« J'ai l'impression que vous et votre partenaire n'avez pas tout à fait la même idée quant à la contraception que vous utilisez :

- *Certaines femmes que je rencontre me disent que leur partenaire leur met de la pression pour qu'elle devienne enceinte. Est-ce que vous vivez cette situation avec votre partenaire ?*
- *Est-ce qu'il vous a déjà menacée ou blessée parce que vous ne vouliez pas devenir enceinte ? »*

Des réponses positives à ces questions vous indiquent que **la femme est susceptible de vivre de la coercition reproductive** sous la forme de *pressions liées à la grossesse.*

Si la femme vous semble évasive ou qu'elle ne veut pas répondre, vous pouvez mettre fin à ces questions et lui remettre la brochure « ***Mon corps, mes choix… Est-ce bien le cas dans votre relation ?*** ».

C'était beaucoup de « si tu m'aimais vraiment, tu voudrais un enfant [avec moi] ».
(Rebecca, 29 ans)[19]

Ça fait que j'ai décidé, encore une fois, de lui faire plaisir parce que je ne voulais pas le perdre. Je me suis pilée dessus et je suis allée me faire avorter.
(Stacey, 26 ans)[20]

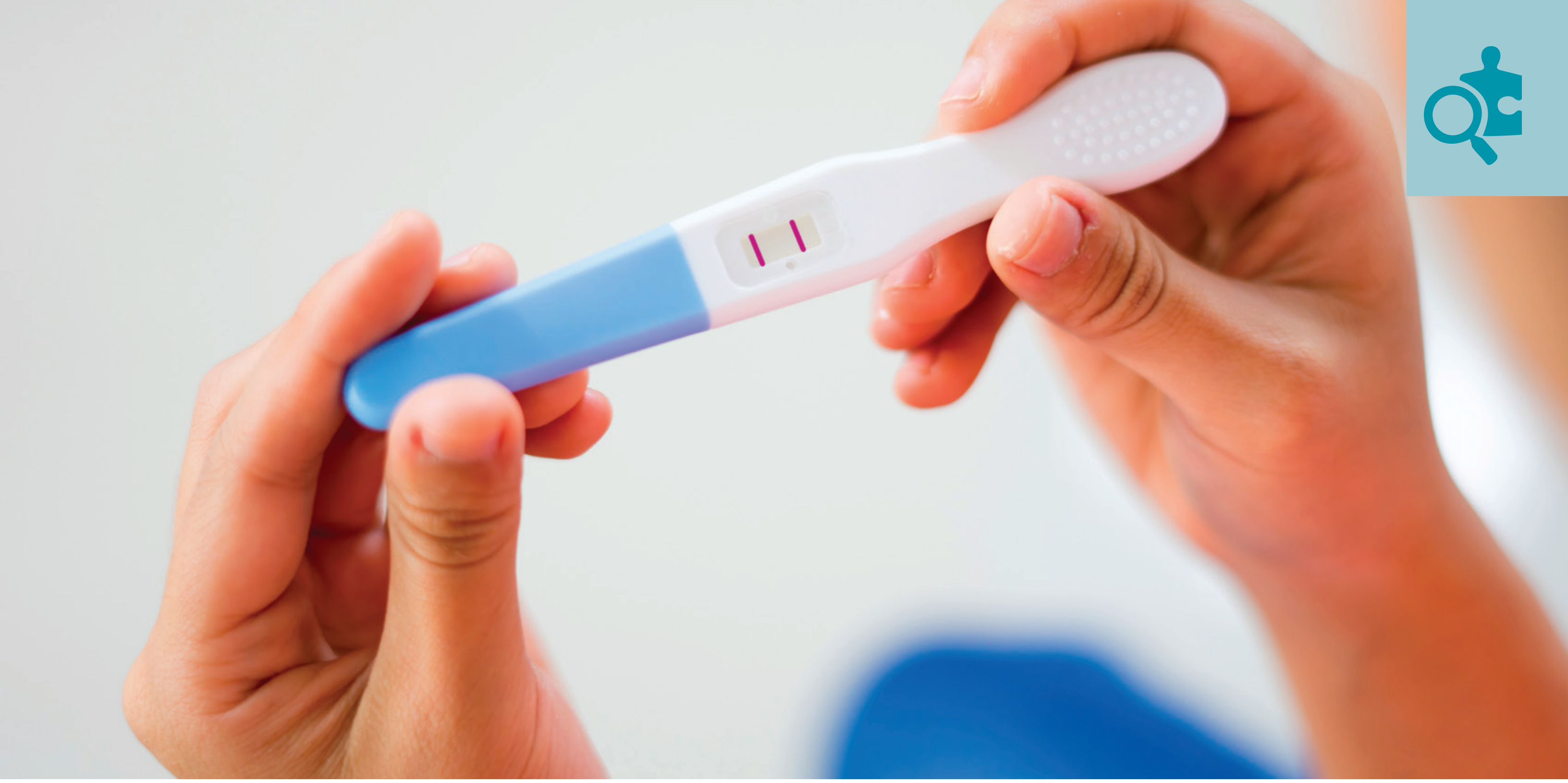

Si la femme est enceinte au moment où vous la rencontrez et qu'elle vous a indiqué vivre de la violence dans le cadre de sa relation avec son partenaire, vous pouvez lui poser ces questions :

« Certaines femmes que je rencontre me disent que leur partenaire n'était pas d'accord avec elles quant à l'issue de la grossesse. Est-ce que votre partenaire était d'accord avec votre choix de [poursuivre ou d'interrompre] *la grossesse ?*

- *Est-ce que votre partenaire vous a déjà obligée à vous faire avorter ?*
- *Est-ce que votre partenaire vous a déjà obligée à poursuivre une grossesse alors que vous ne le souhaitiez pas ?*
- *Est-ce que votre partenaire vous a déjà menacée de vous blesser ou vous a blessée parce que vous ne faisiez pas ce qu'il voulait quant à l'issue de la grossesse ? »*

Des réponses positives à ces questions vous indiquent que la **femme est susceptible de vivre de la coercition reproductive** sous la forme de *coercition lors de la grossesse.*

SOUTENIR

Si la femme dévoile une situation de coercition reproductive ou de violence entre partenaires intimes, il existe différentes façons de la soutenir. Cette prochaine section vous permettra d'identifier des pistes d'intervention pouvant aider la femme et, surtout, de lui démontrer que vous êtes une personne de confiance pouvant la soutenir dans ses démarches.

DÉVOILEMENT DE COERCITION REPRODUCTIVE OU DE VIOLENCE ENTRE PARTENAIRES INTIMES

Lors d'un dévoilement de coercition reproductive ou de violence entre partenaires intimes, **validez les propos de la femme**. Il est important de soutenir que tout acte de violence est condamnable et que l'auteur est le seul responsable des comportements de violence. Vous pouvez tenter de réduire la honte ou la culpabilité qu'elle peut ressentir.

Exemples de réponses :

« Vous avez bien fait de m'en parler. Je suis là pour vous écouter et vous aider. »

« Je suis désolé·e que vous viviez cela. »

« Il arrive parfois qu'on se sente honteuse d'avoir vécu une situation négative, mais sachez que ce n'est pas de votre faute. »

« Il n'y a aucune bonne raison d'exercer de la violence. Les comportements violents sont toujours répréhensibles, même lorsqu'ils sont posés par une personne que l'on aime. »

« Vous n'êtes pas la seule à vivre ce genre de situation. C'est normal de ne pas se sentir bien par rapport à cela. »

La brochure « Mon corps, mes choix.. » invite les femmes à se questionner sur la qualité de leur relation intime. La première section présente des indicateurs d'une relation intime harmonieuse et des exemples de comportements de contrôle ou de violence. La deuxième section aborde la négociation et le contrôle de la contraception. La troisième section illustre les signes de pressions liées à la grossesse. Chacune des sections présente un exemple illustrant des signes de violence ou de coercition reproductive.

Que la femme ait dévoilé ou non une situation de coercition reproductive ou de violence entre partenaires intimes, vous pouvez lui présenter cette brochure d'information. Certaines femmes préféreront la prendre en photo ou la consulter sur place, alors que d'autres préféreront avoir une copie papier. Voici un exemple de scénario qui permet de questionner la femme à ce propos :

« Seriez-vous à l'aise que je vous remette cette brochure d'information ? Certaines personnes préfèrent ne pas prendre de documents, car elles souhaitent éviter que leur partenaire ne les découvre. Si c'est aussi votre situation, faites-moi-le savoir. Vous pourriez prendre en photo la brochure ou la lire sur place. Je pourrais aussi vous l'envoyer en format numérique par courriel. Vous pourrez aussi la consulter en ligne. »

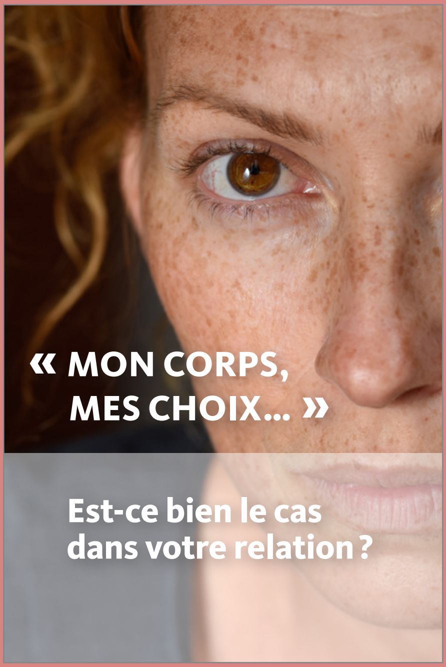

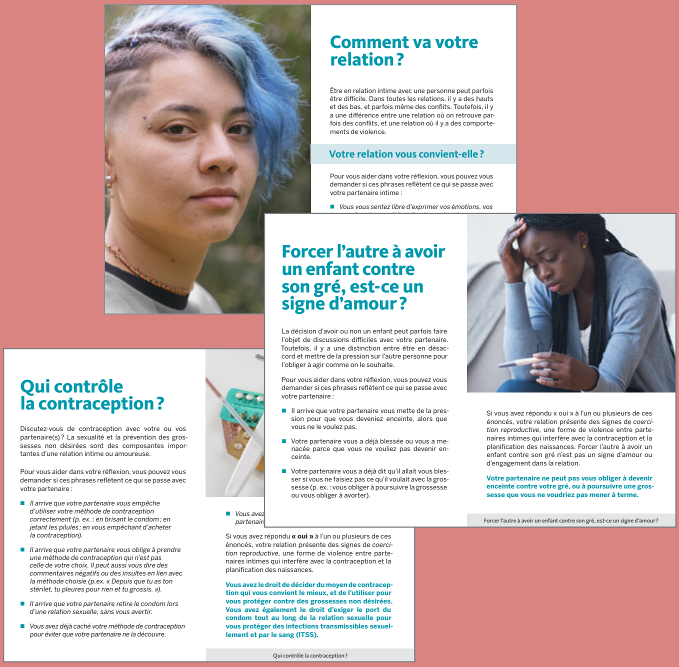

Pourquoi remettre la brochure « Mon corps, mes choix... » aux femmes, même si elles rapportent ne pas être dans une relation violente ?

Les relations changent et évoluent. Il est possible qu'au moment où vous recevez une femme dans votre bureau, sa relation intime ou amoureuse se déroule bien. Toutefois, il est possible que des comportements de violence émergent dans la relation.

Il est possible qu'elle change de partenaire. Ce nouveau partenaire pourrait alors adopter des comportements de violence. Il est aussi possible qu'elle vive de la violence, mais qu'elle ne vous le dévoile pas ou qu'elle ne perçoive pas la situation comme étant de la violence. En lui donnant le document informatif dès maintenant, elle aura déjà l'information lui permettant d'aller chercher de l'aide et du soutien si elle en ressent éventuellement le besoin.

De plus, les femmes et les personnes que vous rencontrez ont des amies, des sœurs, des collègues qui pourraient avoir besoin d'aide. Elles pourront donc les soutenir en leur remettant le document informatif ou en leur spécifiant que des ressources d'aide existent, et que des spécialistes comme vous sont disponibles pour les aider.

Le document informatif permet aussi d'éduquer les femmes sur ces problématiques. Lorsque nous n'avons pas les mots pour nommer ce qui nous arrive ou ce qui arrive à une personne de notre entourage, il est plus difficile d'évaluer la situation et le besoin de soutien. Ce document informatif permet de renseigner les femmes sur les problématiques de la coercition reproductive et de la violence entre partenaires intimes en les informant des ressources d'aide disponibles. D'ailleurs, la littérature démontre que les femmes qui reçoivent ce type d'information seraient plus susceptibles de quitter un partenaire violent, qu'elles aient ou non dévoilé la situation de violence à un·e professionnel·le de la santé ou un·e intervenant·e.

En recevant la brochure informative, les femmes ont l'information sous la main, que ce soit pour elles-mêmes ou pour leurs proches. Elles sauront aussi que vous êtes sensibles à ces questions et qu'elles ont en vous un·e allié·e.

DIRIGER

Si une femme dévoile une situation de coercition reproductive ou de violence entre partenaires intimes, dirigez-la vers des organismes spécialisés en violence ou en santé sexuelle et reproductive.

Certaines personnes apprécieront un soutien concret lors de cette étape : un référencement personnalisé (p. ex. : *« Patricia, de [nom de l'organisme], est disponible pour discuter avec vous. Voici comment vous pouvez la joindre : [numéro de téléphone ou courriel].* »), un référencement en présence (p. ex. : vous faites l'appel et mettez en contact la femme et la personne-ressource de l'organisme ciblé), alors que d'autres préféreront contacter par elles-mêmes l'organisme ou la ressource qu'elles auront choisie, au moment qui leur convient. Offrez-leur le choix.

Vous pouvez aussi informer la femme qu'elle peut consulter, de manière sécuritaire et anonyme, les sites des grands regroupements québécois en matière de violence et de planification des naissances afin d'avoir plus d'informations sur ces questions et de se familiariser avec ces ressources. Vous pouvez aussi lui expliquer qu'il est toujours possible d'effacer un historique de navigation, et lui proposer de lui montrer la procédure au besoin.

Ces ressources peuvent offrir de l'information, du soutien ou de l'accompagnement en fonction des besoins exprimés par les femmes.

Vous pouvez vous-mêmes consulter ces organismes pour avoir davantage d'informations sur les services de soutien ou les services de santé qu'ils offrent.

Pour les **enjeux de sécurité quant à l'accès à des ressources**, voici ce que vous pouvez spécifier à la femme. Si vous avez un suivi à effectuer avec elle, assurez-vous de lui demander à quel numéro ou de quelle façon vous pouvez la joindre, de sorte à minimiser les risques qu'elle pourrait encourir. De la même manière, assurez-vous de la possibilité de laisser un message vocal qu'elle sera la seule à pouvoir écouter.

« S'il arrive que votre partenaire regarde dans votre cellulaire ou lise vos textos, dites-le-moi. On pourra trouver une façon sécuritaire de se contacter sans que votre partenaire ne le sache. Par exemple, je pourrais communiquer avec vous par courriel plutôt que par téléphone. »

Voici une liste non exhaustive de services de santé et d'écoute où vous pourrez diriger les personnes qui vous consultent.

SOS VIOLENCE CONJUGALE
Accessible partout au Québec | 1 800 363-9010

Écoute téléphonique : pour du soutien et de l'information, disponible en anglais et en français, 24h sur 24 et 7 jours sur 7.

GROSSESSE-SECOURS
Accessible partout au Québec, locaux à Montréal
514 271-0554 | Sans frais : 1 877 271-0555

Écoute téléphonique : toute personne préoccupée par une grossesse peut téléphoner du lundi au vendredi de 9h à 17h.

Clavardage en ligne : service de soutien et d'information en ligne pour toute personne préoccupée par une grossesse. Offert du lundi au vendredi de 9h à 16h30.

Test de grossesse et rencontre avec une intervenante : pour les personnes de tout âge qui souhaitent passer un test de grossesse ou rencontrer une intervenante sur place. Il est préférable de fixer un rendez-vous.

VIOLENCE INFO
Capitale Nationale | 418 667-8770

Écoute téléphonique : pour toute personne désirant se renseigner sur la violence conjugale ou partager des difficultés et des préoccupations en lien avec cette problématique de manière confidentielle. Offert du lundi au vendredi de 8h30 à 12h et de 13h à 16h30.

Intervention individuelle : rencontres avec une intervenante pour discuter de son vécu de violence conjugale, mieux comprendre la problématique, améliorer l'estime de soi, et favoriser des relations saines et égalitaires.

Intervention de groupe : espace d'échange et de prise de parole visant à partager les problématiques vécues et à reprendre du pouvoir sur sa vie.

TEL-JEUNES
Accessible partout au Québec | 1 800 263-2266

Écoute téléphonique : 1 800 263-2266. Disponible tous les jours, 24h sur 24 et 7 jours sur 7.

Texto : 514 600-1002. Disponible tous les jours, entre 8h et 22h30.

Clavardage en ligne : Disponible tous les jours, entre 8h et 22h30.

JEUNESSE, J'ÉCOUTE
Accessible partout au Canada | 1 800 668-6868

Jeunesse, J'écoute est accessible 24/7 et offre des services de soutien en français et en anglais.

Écoute téléphonique : 1 800 668-6868. Disponible tous les jours, 24h sur 24 et 7 jours sur 7.

Texto : envoie le mot PARLER au 686868. Disponible tous les jours, 24h sur 24 et 7 jours sur 7.

SOS GROSSESSE
Accessible partout au Québec, locaux dans la Capitale Nationale | 418 682-6222
Sans frais : 1 877 662-9666

Écoute téléphonique : s'adresse à toute personne s'inquiétant d'être enceinte, préoccupée par une grossesse ou ayant vécu une fausse couche. Offert tous les jours de l'année de 9 h à 21 h.

Clavardage en ligne : service de soutien et d'information à toute personne préoccupée par une grossesse.

Test de grossesse et rencontre avec une intervenante : pour les personnes de tout âge qui souhaitent passer un test de grossesse ou rencontrer une intervenante sur place. Il est préférable de fixer un rendez-vous.

REGROUPEMENT QUÉBÉCOIS DES CENTRES D'AIDE ET DE LUTTE CONTRE LES AGRESSIONS À CARACTÈRE SEXUEL (CALACS)
Accessible partout au Québec | 1 888 933-9007

Référence téléphonique : permet de trouver le CALACS le plus près du lieu de résidence. Il est aussi possible d'avoir des rencontres individuelles avec une intervenante qualifiée, de participer à des groupes de soutien, de recevoir de l'information et de l'accompagnement en lien avec le système judiciaire ainsi que de l'information et du soutien pour remplir le formulaire de l'Indemnisation des victimes d'actes criminels (IVAC).

FÉDÉRATION DU QUÉBEC POUR LE PLANNING DES NAISSANCES (FQPN)
En ligne

Référence en ligne : pour en apprendre davantage sur la contraception, les grossesses non planifiées, la fertilité et l'infertilité, la justice reproductive et plus encore, consultez le site Web de la FQPN au fqpn.qc.ca. Vous trouverez aussi sur le site un répertoire de ressources en santé sexuelle et reproductive sur l'ensemble du territoire québécois en cliquant sur l'onglet « trouver de l'aide ». Vous pourrez entre autre trouver une liste des ressources offrant des services d'avortement partout sur le territoire québécois.

INFO-SANTÉ ET INFO-SOCIAL
Accessible partout au Québec | 811

Référence téléphonique : Le volet Info-Santé permet de joindre rapidement une infirmière en cas de problème de santé **non urgent**. Vous pourrez aussi avoir de l'information sur les cliniques offrant des services d'avortement partout sur le territoire québécois. Le volet Info-Social permet de joindre rapidement un·e intervenant·e en cas de problème psychosocial. Au besoin, une ressource appropriée sera proposée dans le réseau de la santé et des services sociaux ou une ressource communautaire. Le service est offert 24 heures par jour, 365 jours par année.

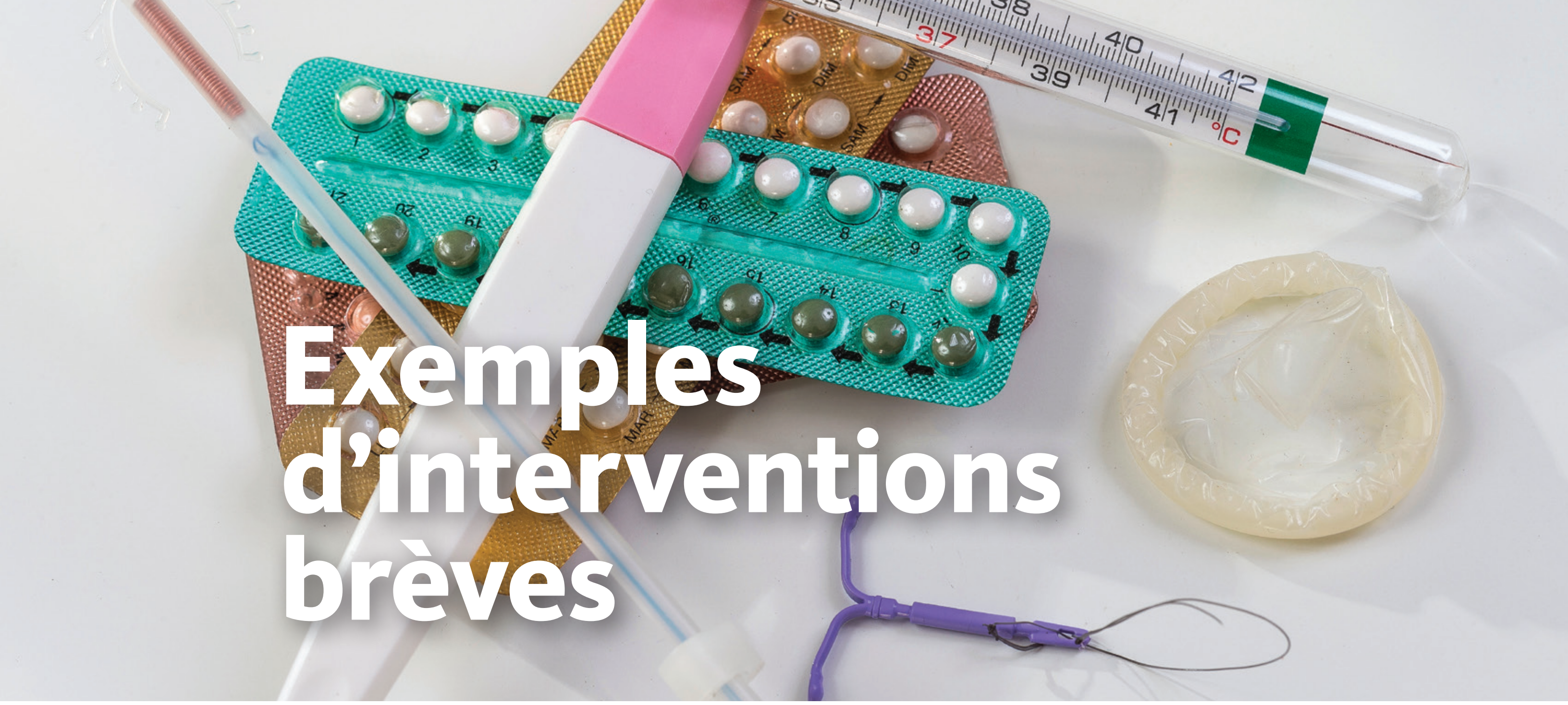

Exemples d'interventions brèves

Lorsqu'une femme qui vous consulte révèle une situation de coercition reproductive où son autonomie reproductive est brimée par son partenaire, vous pouvez lui proposer des méthodes de contraception qui sont peu détectables et sur lesquelles elle pourrait avoir un plus grand contrôle.

La présence de coercition reproductive révèle souvent la présence de violence entre partenaires intimes. Ainsi, nous vous encourageons à diriger la femme vers des organismes spécialisés en violence afin de l'amener à mieux reconnaître la violence et à agir pour favoriser sa sécurité et son bien-être.

Dans une optique de réduction des méfaits, voici des exemples d'interventions selon la situation rapportée.

La femme qui vous consulte dévoile que son partenaire lui **impose une méthode de contraception plutôt qu'une autre** :

« Parfois, les femmes qui me consultent me disent qu'elles ne savent plus quelle méthode de contraception choisir. Leur partenaire les incite à prendre une méthode en particulier, alors que ce n'est pas vraiment leur premier choix. En fait, la meilleure méthode de contraception, c'est celle que l'on choisit pour soi. Vous êtes la seule personne qui peut prendre des décisions sur votre corps. Cela s'applique aussi aux décisions que vous pouvez prendre par rapport à la contraception et aussi à la reproduction. »

La femme qui vous consulte dévoile que son partenaire **limite son usage de la contraception** (p. ex. : retirer le condom sans avertir, jeter les pilules contraceptives, les timbres contraceptifs ou les anneaux vaginaux, etc.) :

« Si vous le souhaitez, nous pourrions regarder ensemble d'autres méthodes de contraception qui vous permettraient d'avoir un plus grand contrôle sur votre santé reproductive. Ces méthodes de contraception sont efficaces et peu détectables par votre partenaire :*

- *Stérilet avec hormones (SIU);*
- *Stérilet sans hormones (DIU);*
- *Injection contraceptive « Depo-Provera »;*
- *Implant contraceptif Nexplanon.*

La femme qui vous consulte est enceinte et son partenaire l'oblige **à poursuivre la grossesse** alors qu'elle n'en a pas envie :

« Décider ou non de poursuivre une grossesse est une décision qui vous appartient. Sachez qu'il existe des ressources où vous pouvez avoir accès à un avortement gratuit et sécuritaire. Le personnel soignant pourra vous dire comment se produit une fausse couche pour que vous puissiez répondre aux questions de votre partenaire s'il vous interroge quant à la fin de la grossesse.

Pour l'accès à l'avortement, voici les ressources disponibles dans votre région... [**à adapter selon votre région**]. *Les ressources peuvent également vous soutenir si vous vivez de l'indécision ou de l'ambivalence face à l'issue de cette grossesse. »*

** Pour plus d'informations sur les méthodes de contraception offertes et leur description, vous pouvez consulter le site Le sexe et moi de la Société des obstétriciens et gynécologues du Canada, ainsi que la section Les méthodes contraceptives sur le site de la Fédération du Québec pour le planning des naissances. »* bas de page

Références

Cette liste présente plusieurs références qui ont été mobilisées pour la conception de ce fascicule. Pour retrouver une ou des références, référez-vous au chiffre indiqué à la suite d'une phrase. La référence associée se trouve ci-dessous.

1. Silverman JG, Raj A. Intimate partner violence and reproductive coercion: global barriers to women's reproductive control. *PLoS Med.* 2014;11(9).
2. Kazmerski T, McCauley HL, Jones K, et al. Use of Reproductive and Sexual Health Services Among Female Family Planning Clinic Clients Exposed to Partner Violence and Reproductive Coercion. *Maternal and Child Health Journal.* 2015;19:1490-1496.
3. Laforest J, Gagné D. Chapitre 5. La violence conjugale. In: Laforest J, Maurice P, Bouchard LM, eds. *Rapport québécois sur la violence et la santé.* Montréal: Institut national de santé publique du Québec; 2018.
4. Burczycka M. *Tendances en matière de violence conjugale autodéclarée au Canada, 2014.* Ottawa, Canada: Statistique Canada;2016.
5. Flores J, Gravel M-A, Lecours C. *Compendium sur la mesure de la violence conjugale au Québec.* Québec: Institut de la statistique du Québec; 2017.
6. Sprague S, Goslings JC, Hogentoren C et al. *Prevalence of intimate partner violence across medical and surgical health care settings : a systematic review.* Violence Against Women. 2014;20(1):118-136.
7. Sinha M. *Family violence in Canada: A statistical profil,* 2011. Ottawa, Canada: Statistique Canada; 2013.
8. Miller E, McCauley HL, Decker MR, et al. Implementation of a family planning clinic–based partner violence and reproductive coercion intervention: Provider and patient perspectives. *Perspectives on sexual and reproductive health.* 2017;49(2):85-93.
9. Zachor H, Chang JC, Zelazny S, Jones KA, Miller E. Training reproductive health providers to talk about intimate partner violence and reproductive coercion: an exploratory study. *Health education research.* 2018;33(2):175-185.
10. Chamberlain L, Levenson R. Addressing Intimate Partner Violence, Reproductive and Sexual Coercion. *A Guide for Obstetric, Gynecologic and Reproductive Health Care Settings, Second Edition San Francisco, CA: Futures Without Violence.* 2012;27.
11. Tancredi DJ, Silverman JG, Decker MR, et al. Cluster randomized controlled trial protocol: addressing reproductive coercion in health settings (ARCHES). *BMC women's health.* 2015;15(1):57.
12. Miller E, Decker MR, McCauley HL, et al. A family planning clinic partner violence intervention to reduce risk associated with reproductive coercion. *Contraception.* 2011;83(3):274-280.
13. Black MC, Basile KC, Breiding MJ, et al. The national intimate partner and sexual violence survey: 2010 summary report. *Atlanta, GA: National Center for Injury Prevention and Control, Centers for Disease Control and Prevention.* 2011;19:39-40.
14. Clark LE, Allen RH, Goyal V, Raker C, Gottlieb AS. Reproductive coercion and co-occurring intimate partner violence in obstetrics and gynecology patients. *American Journal of Obstetrics & Gynecology.* 2014;210(1):e41-48.
15. Sutherland MA, Fantasia HC, Fontenot H. Reproductive coercion and partner violence among college women. *Journal of Obstetric, Gynecologic & Neonatal Nursing.* 2015;44(2):218-227.
16. American College of Obstetricians and Gynecologists. Reproductive and sexual coercion. *Committee on Health Care for Underserved Women.* 2013;554.
17. Nikolajski C, Miller E, McCauley HL, et al. Race and reproductive coercion: a qualitative assessment. *Womens Health Issues.* 2015;25(3):216-223.
18. Moore AM, Frohwirth L, Miller E. Male reproductive control of women who have experienced intimate partner violence in the United States. *Social Science & Medicine* 2010;70(11):1737-1744.
19. Lévesque S, Rousseau C, Dumerchat M. Influence of the relational context on reproductive coercion and the associated consequences *Violence Against Women.* 2020:1-23.
20. Lévesque S, Rousseau C. *La coercition reproductive. Exploration qualitative des expériences de jeunes femmes adultes québécoises.* Montréal : Université du Québec à Montréal; 2019.
21. *Réclamer notre pouvoir et notre place. Le rapport final de l'enquête nationale sur les femmes et les filles autochtones disparues et assassinées.* 2019.
22. Roberts DE. *Killing the black body race, reproduction, and the meaning of liberty.* New York: New York Pantheon Books; 1997.
23. Bridges KM. *Reproducing Race: An Ethnography of Pregnancy as a Site of Racialization.* University of California Press; 2011.
24. Brodsky A. Rape-adjacent: Imagining legal responses to nonconsensual condom removal. *Columbia Journal of Gender and Law.* 2017;32(2):183-210.
25. Grace KT, Anderson JC. Reproductive coercion: A systematic review. *Trauma, Violence, & Abuse.* 2018;19(4):371-390.
26. Holliday CN, Miller E, Decker MR, et al. Racial differences in pregnancy intention, reproductive coercion, and partner violence among family planning clients: A qualitative exploration. *Women's Health Issues.* 2018;28(3):205-211.
27. Jones KA, Cornelius MD, Silverman JG, et al. Abusive experiences and young women's sexual health outcomes: Is condom negotiation self efficacy a mediator ? *Perspectives on Sexual and Reproductive Health.* 2016;48(2):57-64.
28. Albright AE, Allen RS. HPV misconceptions among college students: The role of health literacy. *Journal of Community Health.* 2018;43:1192-1200.
29. Needham HE, Wiemann CM, Tortolero SR, Chacko MR. Relationship between health literacy, reading comprehension, and risk for sexually transmitted infections in young women. *Journal of Adolescent Health.* 2010;46(5):506-508.
30. Miller E, Decker MR, McCauley HL, et al. Pregnancy coercion, intimate partner violence and unintended pregnancy. *Contraception.* 2010;81(4):316-322.
31. Samankasikorn W, Alhusen J, Yan G, Schminkey DL, Bullock L. Relationships of reproductive coercion and intimate partner violence to unintended pregnancy. *Journal of Obstetric, Gynecologic, & Neonatal Nursing.* 2019;48(1):50-58.
32. Francis JKR, Malbon K, Braun-Courville D, Linares LO, Rosenthal SL. Relationship between depressive symptoms and birth control sabotage in adolescent females initiating contraception. *Journal of Adolescent Health.* 2015;56(2):S97-S98.
33. Okeke-Ihejirika P, Yohani S, Muster J, Ndem A, Chambers T, Pow V. A Scoping Review on Intimate Partner Violence in Canada's Immigrant Communities. *Trauma, Violence & Abuse.* 2018:1-23.
34. Vissandjée B, Short WE, Bates K. Health and legal literacy for migrants: twinned strands woven in the cloth of social justice and the human right to health care. *BMC International Health and Human Rights* 2017;17(10):1-12.
35. Ending Violence. Association of BC. *Provincial briefing document 4: Executive summary.* British Columbia, Canada: Safety of Immigrant, Refugee, & Non-Status Women Project;2010.
36. Hulme J, Dunn S, Guilbert E, Soon J, Norman W. Obstacles et facilité d'accès à la planification familiale au Canada. *Healthcare Policy.* 2015;10(3).
37. BC Provincial Mental Health and Substance Use Planning Council. *Trauma-Informed Practice Guide.* British Columbia, Canada2013.
38. Purkey E, Patel R, Phillips SP. Trauma-informed care: Better care for everyone. *Canadian Family Physician.* 2018;64(3):170-172.
39. Elliott DE, Bjelajac P, Fallot RD, Markoff LS, Reed BG. Trauma-informed or trauma-denied: principles and implementation of trauma-informed services for women. *Journal of Community Psychology.* 2005;33(4):461-477.
40. Lévesque S, Rousseau C, Pétrieux C. Mieux répondre aux besoins en santé sexuelle et reproductive des femmes ayant vécu de la coercition reproductive par une approche de soins sensibles aux traumas: Résultats d'une étude qualitative exploratoire. In: Trajet-vi, ed. *Pratiques et recherches féministes en matière de violence cojnugale - Co-construction des connaissances et expertises.* Montréal, Canada: Presses de l'Université du Québec; sous presse.
41. Beagan BL. Approaches to culture and diversity: A critical synthesis of occupational therapy literature. *Canadian Journal of Occupational Therapy.* 2015;82(5):272-282.
42. Baba L. *Sécurité culturelle en santé publique chez les premières nations, les inuits et les métis. État des lieux sur la compétence et la sécurité culturelles en éducation, en formation et dans les services de santé.* Colombie-Britannique, Canada: Centre de collaboration nationale de la santé autochtone (CCNSA);2013.
43. Kinébanian A, Stomph M. Diversity matters: guiding principles on diversity and culture. *World Federation of Occupational Therapists Bulletin.* 2010;61(1):5-13.
44. Anderson J, Browne A, Basu Khan K, Lynam J. « Rewriting » cultural safety within the postcolonial and postnational feminist project. Toward new epistemologies of healing. *Advances in Nursing Science.* 2003;26(3):196-214.
45. Mullaly B. *Challenging Oppression and Confronting Privilege.* 2 ed. Ontario: Oxford University Press; 2010.
46. Lowik AJ. *Services d'avortement adaptés aux réalités trans: un guide visant à permettre aux prestataires de soins d'instaurer des politiques et des pratiques qui tiennent compte des réalités trans dans un contexte d'avortement.* Montréal: Fédération du Québec pour le planning des naissances;2017.
47. Curtis E, Jones R, Tipene-Leach D, et al. Why cultural safety rather than cultural competency is required to achieve health equity: a literature review and recommended definition. *International Journal of Equity in Health.* 2019;18(174):1-17.
48. Jull JE, Giles AR. Health equity, Aboriginal peoples and occupational therapy. *Canadian Journal of Occupational Therapy.* 2012;79:70-76.
49. National Aboriginal Health Organization (NAHO). *Cultural competency and safety: A guide for health care administrations, providers and educators.* Ottawa, Ontario;2008.
50. Milot T, Lemieux R, Berthelot N, Collin-Vézina D. Les pratiques sensibles au trauma. In: Milot T, Collin-Vézina D, Godbout N, eds. *Trauma complexe. Comprendre, évaluer et intervenir.* Québec: Presses de l'Université du Québec; 2018:269-285.

Pour en savoir plus

Pour en apprendre davantage sur l'**approche de soins sensibles aux traumas**

Elliott, D. E., Bjelajac, P., Fallot, R. D., Markoff, L. S. et Reed, B. G. (2005). Trauma-informed or trauma-denied: principles and implementation of trauma-informed services for women. *Journal of Community Psychology*, *33*(4), 461-477.

Milot, T., Lemieux, R., Berthelot, N. et Collin-Vézina, D. (2018). Les pratiques sensibles au trauma. Dans T. Milot, D. Collin-Vézina et N. Godbout (dir.), *Trauma complexe. Comprendre, évaluer et intervenir* (p. 269-285). Québec : Presses de l'Université du Québec.

Zachor, H., Chang, J. C., Zelazny, S., Jones, K. A. et Miller, E. (2018, Apr 1). Training reproductive health providers to talk about intimate partner violence and reproductive coercion: an exploratory study. *Health Educ Res*, *33*(2), 175-185.

Pour en apprendre davantage sur la **sécurisation culturelle**

Baba, L. (2013). *Sécurité culturelle en santé publique chez les premières nations, les inuits et les métis. État des lieux sur la compétence et la sécurité culturelles en éducation, en formation et dans les services de santé*. Colombie-Britannique, Canada : Centre de collaboration nationale de la santé autochtone (CCNSA). Récupéré de https://www.ccnsa-nccah.ca/docs/emerging/RPT-CulturalSafetyPublicHealth-Baba-FR.pdf

Beagan, B. L. (2015). Approaches to culture and diversity: A critical synthesis of occupational therapy literature. *Canadian Journal of Occupational Therapy*, *82*(5), 272-282.

Pour en apprendre davantage sur la violence conjugale
Violence conjugale : connaître, détecter, intervenir
Formation offerte en ligne par le ministère de la Santé et des Services sociaux

La formation en violence conjugale est offerte au personnel travaillant dans le réseau, ainsi qu'aux professionnel·le·s de la santé et des services sociaux travaillant hors réseau. Pour faire une demande d'accès à la formation, connectez-vous sur ENA (Environnement numérique d'apprentissage provincial).

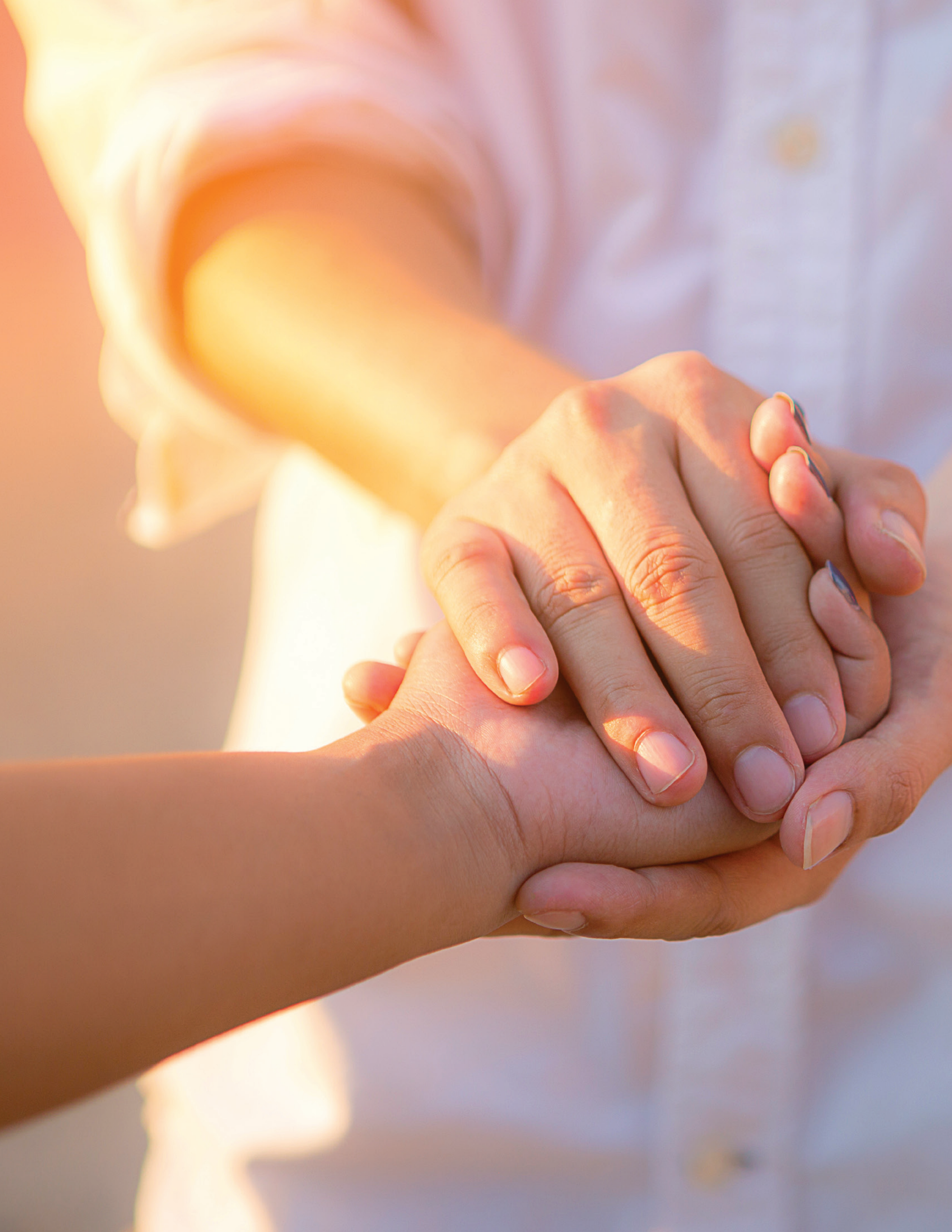

© Sylvie Lévesque, UQAM
