# Supplementary material for: Canadian service providers' perspectives on reproductive coercion and abuse: a participatory action research to address their needs and support their actions
Source: Reprod Health. 2023 Jun 30;20:100. doi: 10.1186/s12978-023-01640-w (PMC10311789; doi:10.1186/s12978-023-01640-w)
Supplement: Supplementary file 2 — Additional file 2. Booklet (French). [file 12978_2023_1640_MOESM2_ESM.pdf]

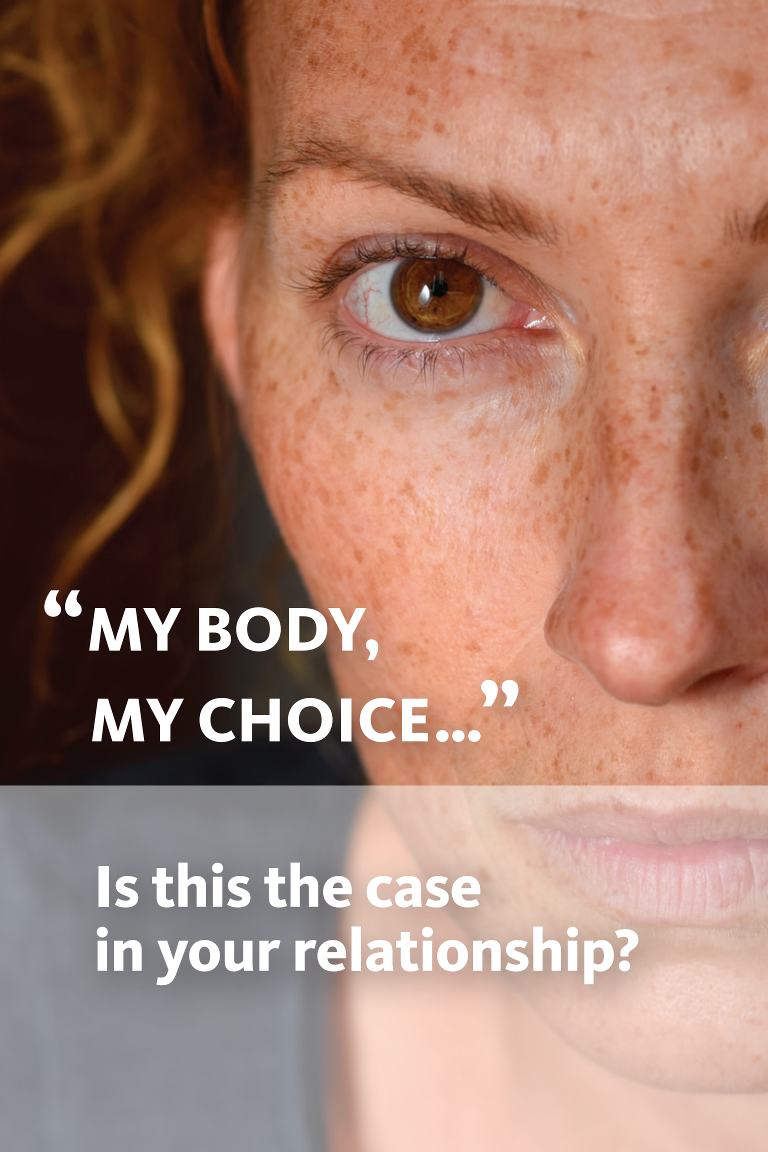
"MY BODY,
MY CHOICE..."
Is this the case
in your relationship?

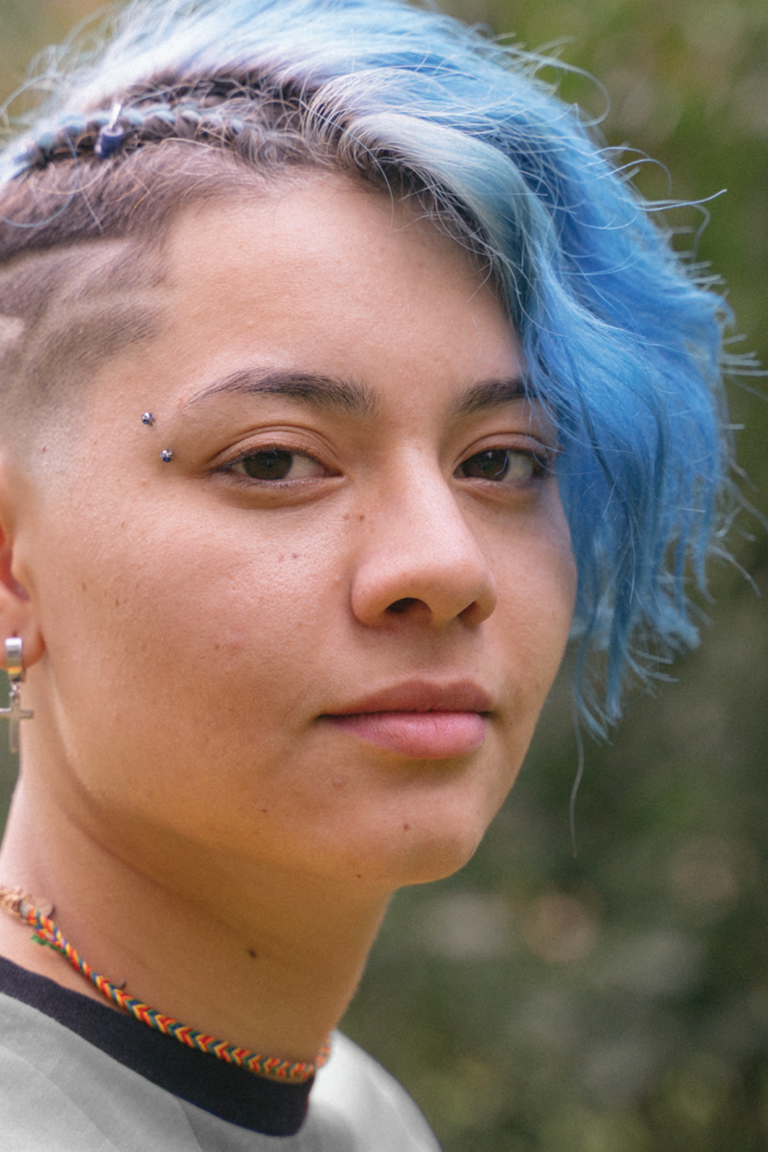

How is Your Relationship Going?

Being in an intimate relationship can sometimes be challenging. Every interpersonal relationship has its share of ups and downs, sometimes even conflict. But there is a difference between a relationship where there is sometimes conflict, and a relationship where there is abusive or violent behaviour.

Is Your Relationship Right for You?

To help you reflect on your relationship, you may ask yourself whether these statements reflect your situation with your intimate partner:

- *You feel free to share your feelings, thoughts, and opinions with your partner.*
- *You feel that your partner respects your boundaries.*
- *When you're not feeling well, you know your partner is there to support you and to help you find solutions.*

If these sentences are representative of your relationship, it seems that you are in a relationship based on care and listening, which reduces your chances of experiencing intimate partner violence.

Does Your Relationship Meet Your Needs?

You can also ask yourself the following questions to better understand your relationship:

- *Does your partner ever control where you go, who you talk to, and how you spend your money? For example, does your partner force you to stop talking to a friend, or to stop going to a place you enjoy?*
- *Does your partner ever humiliate you in private or in front of other people? Do they insult you and put you down?*
- *Does your partner ever hurt you or threaten you, or pressure you into having sex when you don't feel like it?*

If you answered "yes" to one or more of these questions, your relationship is showing signs or behaviours of violence.

Your partner should not control where you go, who you talk to, or how you spend your money. They also cannot force you to have sex or belittle you when they are angry. These behaviours are not acts of love, but of violence.

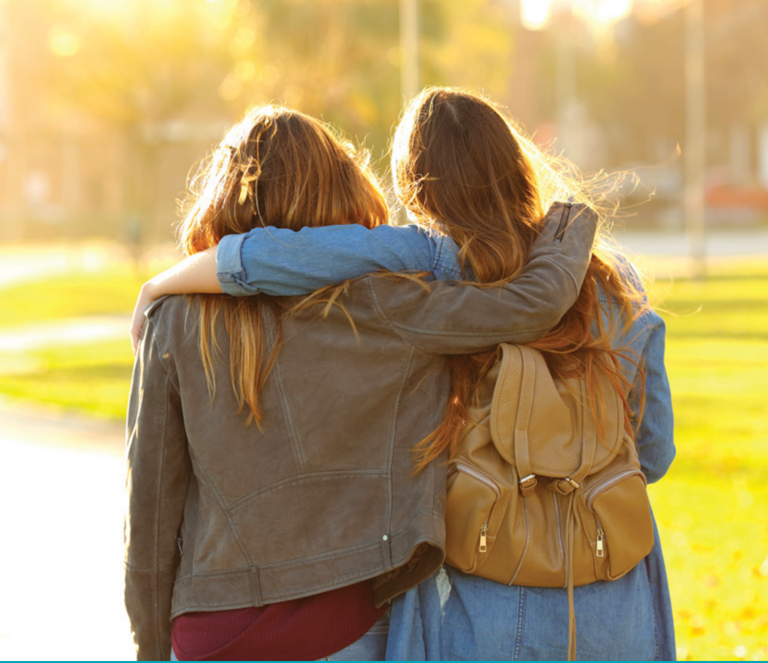

You may feel that this does not apply to you. But while reading this booklet, take the opportunity to think about your relationship. If this information doesn't apply to your situation, it might be able to help a friend. You can give your friend this booklet and encourage them to seek help.

Example of a situation showing signs of intimate partner violence

Abusive relationships are not necessarily violent all the time. In addition, violent behaviour in intimate relationships encompasses more than just physical violence.

Here is an example of a situation where violence is manifested as *control* and *psychological abuse*.

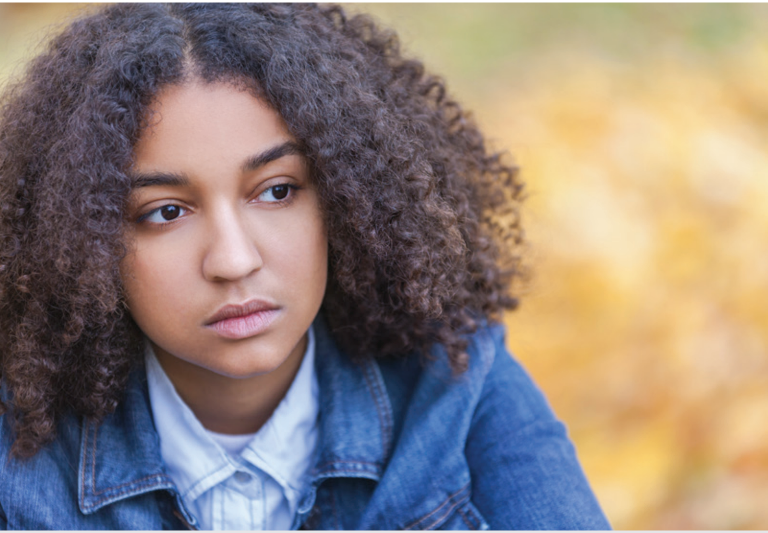

Eli has been in a relationship with Sam for a few months. They like each other very much. They have common interests and understand each other well. Eli has several friends she likes to see. But Sam started telling her that they don't spend enough time together because Eli always hangs out with her friends. The other day, while they were out grocery shopping, Eli got a call from a friend and chatted for a couple of minutes. Sam looked angry. When they got to the cash, Sam insulted Eli, telling her that she was "a jerk who only thought about herself". Sam stormed out of the grocery store, leaving her alone and humiliated at the cash.

If this situation feels familiar to you, or if someone you know is going through a similar situation, don't hesitate to talk to a health professional or support provider (resources are listed at the end of this booklet). This person will be able to listen, support and direct you to specialized organizations if necessary.

Who Controls Contraception?

Do you discuss contraception with your partner(s)? Sexuality and preventing unwanted pregnancies are important topics in an intimate or romantic relationship.

To help you reflect on your relationship, you may ask yourself whether these statements reflect your situation with your intimate partner:

- *Your partner prevents you from using your contraceptive method correctly (e.g., tearing the condom, throwing birth control pills away, preventing you from buying contraception).*
- *Sometimes, your partner forces you to take a birth control method that is not the one you prefer. Your partner also insults or speaks negatively of you/ your choice of contraception (e.g., "Since you've been on the pill, you cry for nothing and you're getting fat!").*
- *Sometimes, your partner removes the condom during sex without telling you.*
- *You've hidden your preferred birth control method from your partner.*
- *Sometimes, you're afraid to ask your partner to use a condom.*

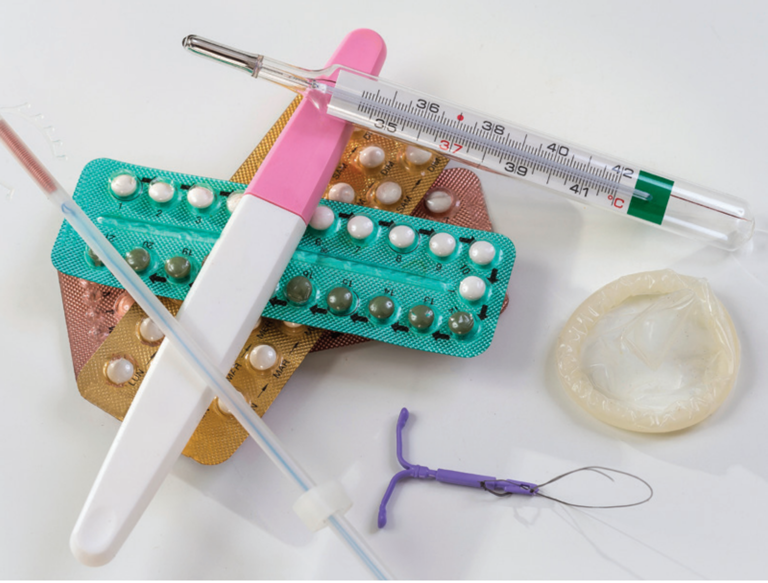

If you answered "yes" to one or more of these statements, your relationship shows signs of reproductive coercion, a form of intimate partner violence that interferes with contraception and family planning.

You have the right to decide which method of contraception is best for you, and to use it to protect yourself against unwanted pregnancy. You also have the right to require that your partner wears a condom during sex to protect yourself from sexually transmitted infections (STIs).

Example of a situation that shows signs of contraceptive sabotage

Taking contraception is a personal decision that helps prevent unwanted pregnancies and STIs. When a partner prevents you from using contraception the way you want, it is referred to as *contraceptive sabotage*.

In this example, Evelyn's partner doesn't necessarily want her to get pregnant. Rather, he wants to control their sexual relationship in a way that increases his own sexual pleasure, without regard for Evelyn's sexual and reproductive health.

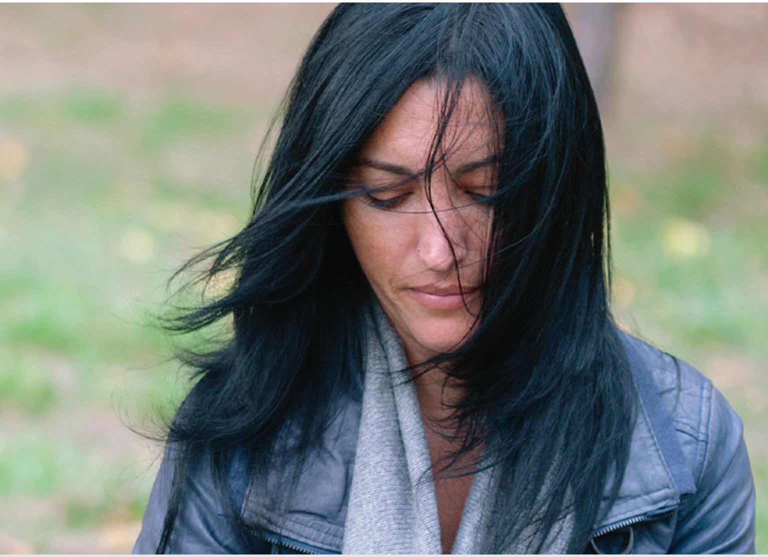

Evelyn has had a new partner for a few months. They are not a couple, but they occasionally have sex. Evelyn doesn't want to get pregnant, so she uses a condom during sex. However, she feels that every time they have sex, she has to convince him to use a condom. He usually agrees to use it, but reluctantly. He often tells her that he finds sex less pleasurable with a condom. The last time they had sex, Evelyn realized that he'd removed the condom without telling her. Now she's worried about having an STI or becoming pregnant.

If this situation feels familiar to you, or if someone you know is going through a similar situation, don't hesitate to talk to a health professional or support provider (resources are listed at the end of this booklet). Taking contraception and using a condom are personal decisions that help prevent unwanted pregnancies and STIs. A healthcare professional or support provider can tell you about contraceptive methods that are difficult for your partner to detect (e.g., an intrauterine device, also known as IUD). They can also put you in touch with a professional who is familiar with the type of situation you're experiencing.

Forcing Someone to Have a Child Against Their Will: An Act of Love?

The decision to have (or not to have) a child can sometimes be the subject of difficult conversations with your partner. However, there is an important difference between disagreeing and pressuring the other person to make them act the way you want them to.

To help you reflect on your relationship, you may ask yourself whether these statements reflect your situation with your intimate partner:

- Sometimes, your partner puts pressure on you to get pregnant when you don't want to.
- Your partner has hurt or threatened you because you didn't want to get pregnant.
- Your partner has told you that they would hurt you if you don't do what they want with the pregnancy (e.g., forcing you to continue with the pregnancy or forcing you to have an abortion).

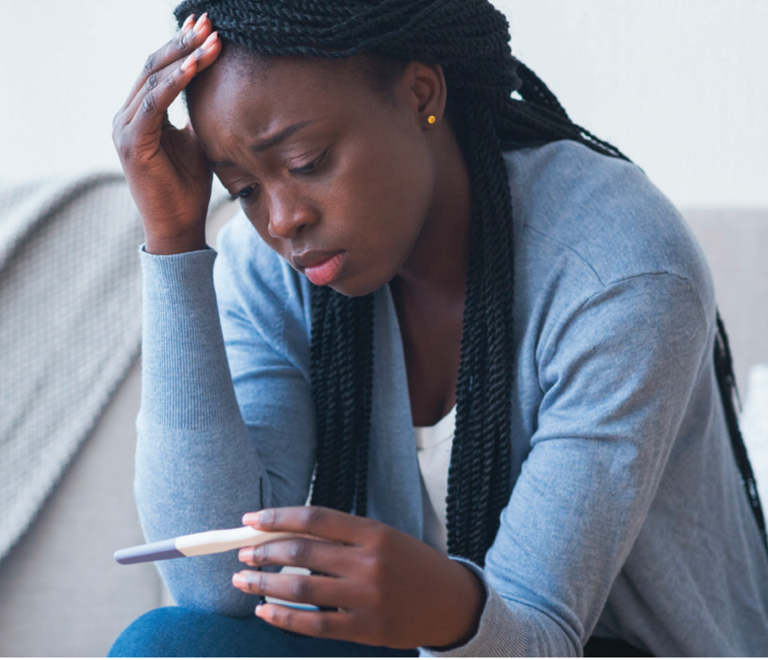

If you answered “yes” to one or more of these statements, your relationship shows signs of reproductive coercion, a form of intimate partner violence that interferes with contraception and family planning. Forcing a partner to have a child against their will is not an act of love or commitment to the relationship.

Your partner cannot force you to become pregnant against your will, or to continue a pregnancy that you do not wish to carry to term.

Example of a situation of exerted pressure related to pregnancy and manifestations of psychological violence

Intimate and romantic relationships change over time. Sometimes, new situations or new discussions create conflict for the couple. It is possible for these conflicts to become increasingly violent. This example reveals situations of *psychological violence* and *pressure related to pregnancy* by Miguel against his partner Anna.

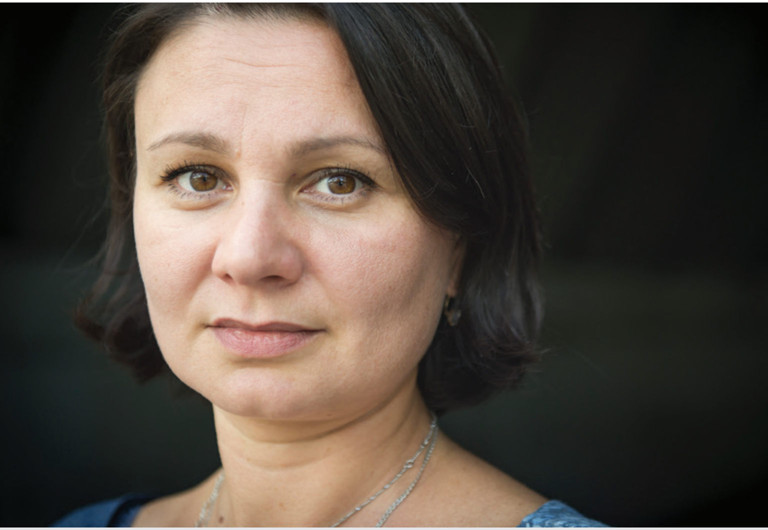

Anna has two children from a previous marriage. She has been in a relationship with Miguel for three years. For several months now, Miguel has been telling Anna that he wants to have a child with her. Anna has told Miguel that she doesn't want to have more children. But Miguel persisted, becoming more and more insistent. When Anna meets a support provider at a community organization, she confides in her that Miguel often tells her: "If you really loved me, you would want to have a child with me. So I take it you don't love me as much as I love you." Anna feels stuck.

If this situation feels familiar to you, or if someone you know is going through a similar situation, don't hesitate to talk to a health professional or support provider (resources are listed at the end of this booklet). This person will be able to listen, support and—if you wish—direct you to specialized resources.

To inquire or seek help

If you need to talk, ask questions, or want access to support or healthcare services (STIs screening, pregnancy test, contraception and emergency contraception, etc.), resources are available. Services are free and confidential.

SOS DOMESTIC VIOLENCE

Accessible everywhere in Quebec | 1 800-363-9010

Helpline: For support and information, available in English and French, 24/7.

GROSSESSE-SECOURS

Accessible everywhere in Quebec. Office in Montreal
514-271-0554 | Toll-free: 1 877-271-0555

Helpline: Anyone concerned about a pregnancy can call, from Monday to Friday, 9 a.m. to 5 p.m.

Online chat: Online support and information for anyone concerned about pregnancy. Available Monday to Friday, 9 a.m. to 4:30 p.m.

Pregnancy test and meeting with a support provider: For people of all ages who want to take a pregnancy test or meet a support provider on site. It is recommended to make an appointment.

SOS GROSSESSE

Accessible everywhere in Quebec. Office in Quebec City
418-682-6222 | Toll-free: 1 877-662-9666

Helpline: For anyone concerned about pregnancy or having experienced a miscarriage. Available daily from 9 a.m. to 9 p.m.

Online chat: Support and information for anyone concerned about pregnancy.

Pregnancy test and meeting with a support provider: For people of all ages who want to take a pregnancy test or meet a support provider on site. It is recommended to make an appointment.

VIOLENCE INFO

Capitale Nationale | 418-667-8770

Helpline: For anyone seeking information about domestic violence or seeking to discuss their concerns in a confidential manner. From Monday to Friday, 8:30 a.m. to 12 p.m., and from 1 p.m. to 4:30 p.m.

Individual intervention: Consultation with a support provider to discuss domestic violence experiences, better understand the problem, improve self-esteem, and develop healthy and egalitarian relationships.

Group intervention: A safe space to share experiences and regain control over one's life.

TEL-JEUNES

Accessible everywhere in Quebec | 1 800-263-2266

Tel-Jeunes allows all young people 20 years and under in Quebec to talk to a support provider about their concerns.

Helpline: 1 800-263-2266. Available 24/7.

Text: 514-600-1002. Available daily, 8 a.m. to 10:30 p.m.

Online chat: Available daily, 8 a.m. to 10:30 p.m.

KIDS HELP PHONE

Accessible everywhere in Canada | 1 800-668-6868

Kids Help Phone is accessible 24/7 and offers support services in French and English.

Helpline: 1 800-668-6868. Available every day, 24/7.

Text: Text the word CONNECT to 686868. Available 24/7.

REGROUPEMENT QUÉBÉCOIS DES CENTRES D'AIDE ET DE LUTTE CONTRE LES AGRESSIONS À CARACTÈRE SEXUEL (CALACS)

Accessible everywhere in Quebec | 1 888-933-9007

Phone reference: An organization grouping sexual assault help centres in Quebec that allows you to find the CALACS centre closest to your place of residence. It is also possible to schedule individual meetings with a professional worker, to participate in support groups, to receive information and support related to the justice system and/or help completing the Crime Victims' Compensation (IVAC) form.

FÉDÉRATION DU QUÉBEC POUR LE PLANNING DES NAISSANCES (FQPN) | Online

Online reference: To learn more about contraception, unplanned pregnancies, fertility and infertility, reproductive justice and more, visit the FQPN website at fqpn.qc.ca.

The site also features a directory of sexual and reproductive health resources in Quebec, including a list of resources offering abortion services, by clicking under the tab "Trouver de l'aide".

INFO-SANTÉ AND INFO-SOCIAL

Accessible everywhere in Quebec | 811

Phone reference: Info-Santé makes it possible to quickly reach a nurse in the event of a non-urgent health problem. You can also obtain information on clinics offering abortion services throughout Quebec. Info-Social allows you to quickly reach a support provider in the event of a psychosocial problem. If necessary, Info-Social will direct you to an appropriate resource in the healthcare or social services network, or to a resource in the community. The service is offered 24 hours a day, 365 days a year.

This booklet complements the *Coercition reproductive et violence entre partenaires intimes* guide intended for health professionals and support providers. Translation of this booklet was made possible through a grant from *Équipe VC : Acteurs en contexte et pratiques novatrices*.

© Sylvie Lévesque, UQAM

Legal deposit
Bibliothèque et Archives nationales du Québec, 2021
ISBN : 978-2-9818518-3-3
