# Supplementary material for: Canadian service providers' perspectives on reproductive coercion and abuse: a participatory action research to address their needs and support their actions
Source: Reprod Health. 2023 Jun 30;20:100. doi: 10.1186/s12978-023-01640-w (PMC10311789; doi:10.1186/s12978-023-01640-w)
Supplement: Supplementary file 3 — Additional file 3. Booklet (English). [file 12978_2023_1640_MOESM3_ESM.pdf]

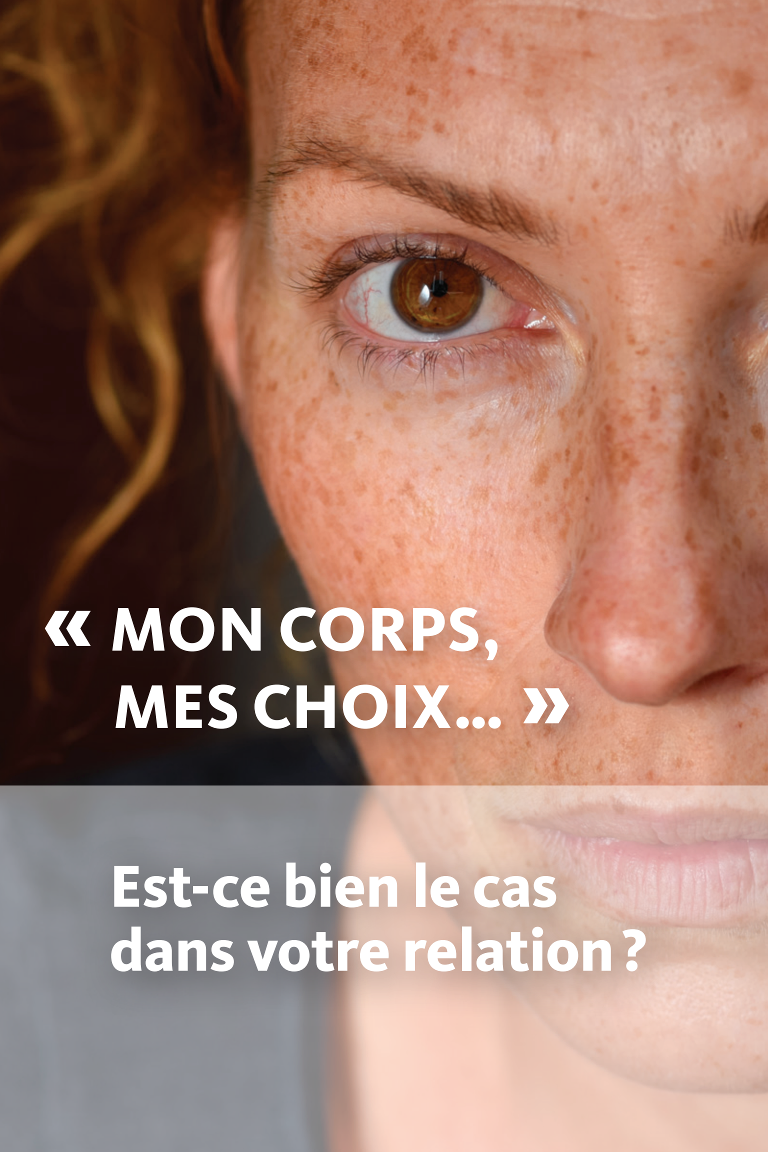

« MON CORPS, MES CHOIX... »

Est-ce bien le cas dans votre relation ?

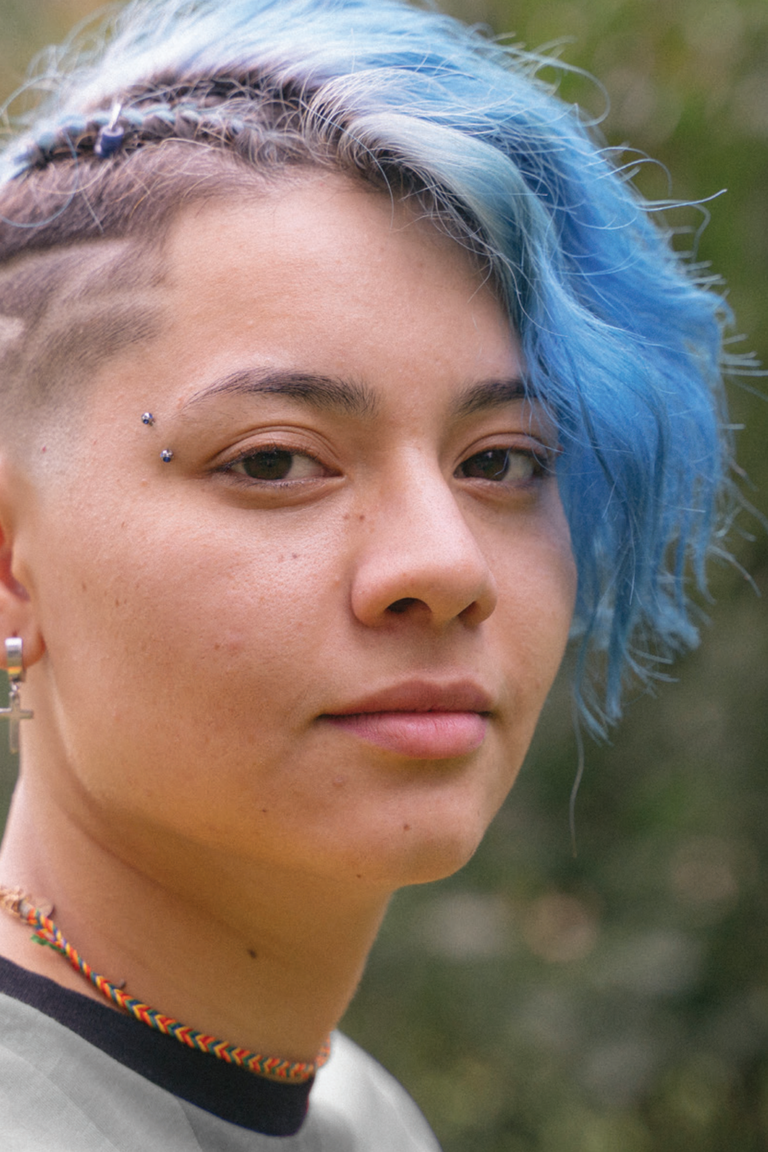

Comment va votre relation ?

Être en relation intime avec une personne peut parfois être difficile. Dans toutes les relations, il y a des hauts et des bas, et parfois même des conflits. Toutefois, il y a une différence entre une relation où on retrouve parfois des conflits, et une relation où il y a des comportements de violence.

Votre relation vous convient-elle ?

Pour vous aider dans votre réflexion, vous pouvez vous demander si ces phrases reflètent ce qui se passe avec votre partenaire intime :

- *Vous vous sentez libre d'exprimer vos émotions, vos pensées et vos opinions à votre partenaire.*
- *Vous sentez que votre partenaire respecte vos limites.*
- *Quand vous n'allez pas bien, vous savez que votre partenaire est là pour vous soutenir et vous aider à trouver des solutions.*

Si ces phrases représentent bien votre relation intime, il semble que vous soyez dans une relation où l'entraide et l'écoute sont présentes, ce qui diminue les risques de vivre de la violence.

Votre relation correspond-elle à vos besoins ?

Vous pouvez aussi vous poser les questions suivantes pour mieux comprendre votre relation.

- *Est-ce qu'il arrive que votre partenaire contrôle où vous allez, à qui vous parlez, et comment vous dépensez votre argent (p. ex. : il vous oblige à ne plus parler à un·e de vos ami·e·s, à ne plus aller à un endroit que vous appréciez) ?*
- *Est-ce qu'il arrive que votre partenaire vous humilie en privé ou devant d'autres personnes (p. ex. : vous insulte, vous rabaisse) ?*
- *Est-ce qu'il arrive que votre partenaire vous blesse ou menace de le faire, ou encore met de la pression pour avoir des relations sexuelles alors que vous n'en avez pas envie ?*

Si vous avez répondu « oui » à l'une ou plusieurs des questions ci-dessus, votre relation présente des *signes ou des comportements de violence*.

Votre partenaire ne peut pas contrôler où vous allez, à qui vous parlez ou comment vous dépensez votre argent. Il ne peut pas non plus vous forcer à avoir des relations sexuelles ou vous dénigrer lorsqu'il est fâché. Ces comportements ne sont pas des preuves d'amour, mais des signes de violence.

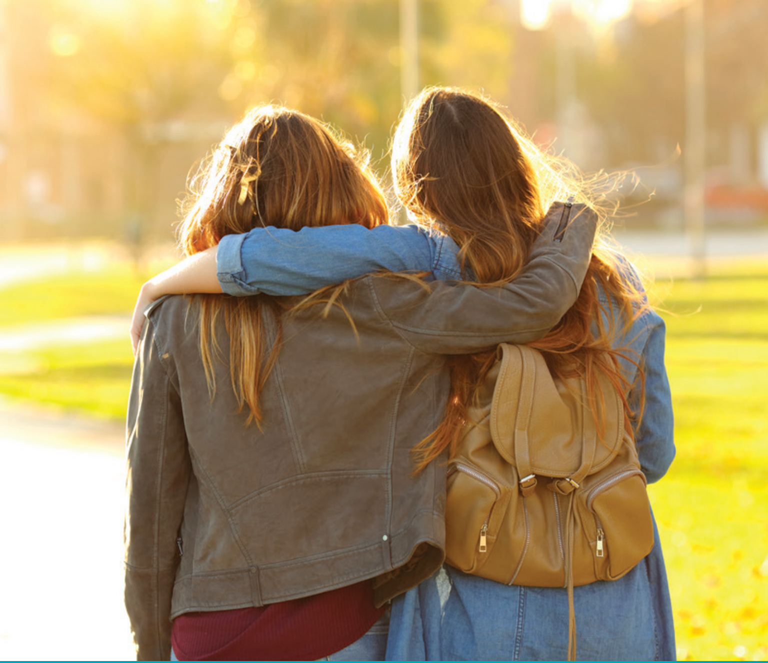

Il est possible que vous ayez l'impression que cela ne vous concerne pas. Profitez tout de même de la lecture de cette brochure pour réfléchir à votre relation. Si son contenu ne s'applique pas à votre situation, il pourrait peut-être aider une amie. Vous pourrez lui remettre cette brochure, en lui disant qu'elle peut recevoir de l'aide.

Exemple de situation qui présente des signes de violence au sein d'une relation intime

Les relations qui présentent de la violence ne sont pas nécessairement violentes en tout temps. De plus, les comportements de violence dans les relations intimes ne se résument pas à la violence physique.

Voici un exemple de situation où il est question de *contrôle* et de *violence psychologique*.

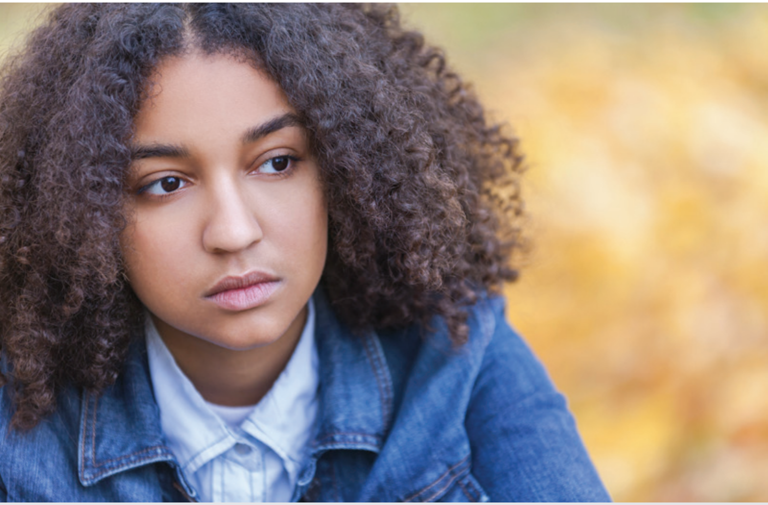

Frédérique est en couple avec Samuel depuis quelques mois. Les deux s'apprécient beaucoup : ils ont des intérêts communs et se comprennent bien l'un et l'autre. Frédérique a plusieurs ami·e·s avec qui elle aime faire des activités. Toutefois, Samuel a commencé à lui dire qu'ils ne passent pas assez de temps ensemble parce que Frédérique voit toujours ses ami·e·s. L'autre jour, alors qu'ils faisaient l'épicerie, Frédérique a reçu un appel d'un ami avec qui elle a discuté quelques minutes. Après l'appel, Samuel semblait en colère. Rendus à la caisse, Samuel a injurié Frédérique en lui disant qu'elle n'était « qu'une conne qui ne pensait qu'à elle ». Samuel a quitté l'épicerie en l'insultant, laissant Frédérique seule à la caisse, humiliée.

Si cette situation ressemble à celle que vous vivez, ou si une personne de votre entourage vit une situation similaire, n'hésitez pas à en parler à un·e professionnel·le de la santé ou un·e intervenant·e (des ressources sont disponibles à la fin du livret). Cette personne pourra vous soutenir, vous écouter, vous aider et vous diriger vers des organismes spécialisés au besoin.

Qui contrôle la contraception ?

Discutez-vous de contraception avec votre ou vos partenaire(s) ? La sexualité et la prévention des grossesses non désirées sont des composantes importantes d'une relation intime ou amoureuse.

Pour vous aider dans votre réflexion, vous pouvez vous demander si ces phrases reflètent ce qui se passe avec votre partenaire :

- *Il arrive que votre partenaire vous empêche d'utiliser votre méthode de contraception correctement (p. ex. : en brisant le condom ; en jetant les pilules ; en vous empêchant d'acheter la contraception).*
- *Il arrive que votre partenaire vous oblige à prendre une méthode de contraception qui n'est pas celle de votre choix. Il peut aussi vous dire des commentaires négatifs ou des insultes en lien avec la méthode choisie (p.ex. « Depuis que tu as ton stérilet, tu pleures pour rien et tu grossis. »).*
- *Il arrive que votre partenaire retire le condom lors d'une relation sexuelle, sans vous avertir.*
- *Vous avez déjà caché votre méthode de contraception pour éviter que votre partenaire ne la découvre.*

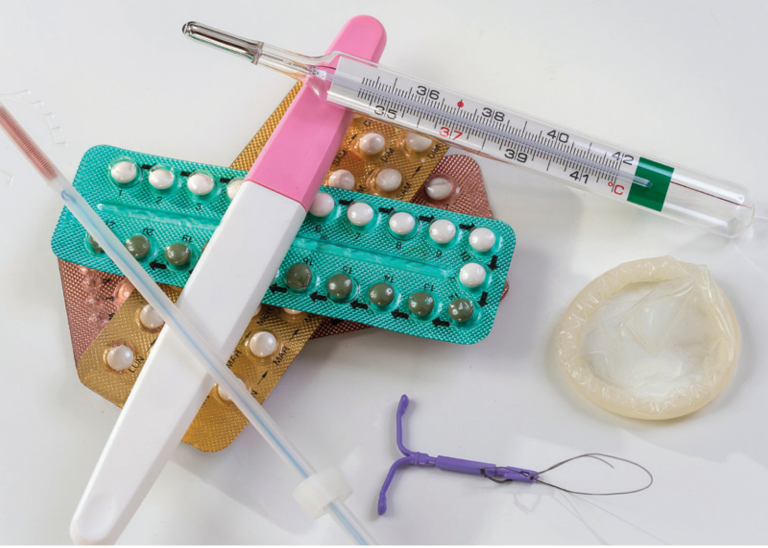

- *Vous avez parfois peur de demander à votre partenaire de mettre un condom.*

Si vous avez répondu « oui » à l'un ou plusieurs de ces énoncés, votre relation présente des signes de *coercition reproductive*, une forme de violence entre partenaires intimes qui interfère avec la contraception et la planification des naissances.

Vous avez le droit de décider du moyen de contraception qui vous convient le mieux, et de l'utiliser pour vous protéger contre des grossesses non désirées. Vous avez également le droit d'exiger le port du condom tout au long de la relation sexuelle pour vous protéger des infections transmissibles sexuellement et par le sang (ITSS).

Exemple de situation qui présente des signes de sabotage contraceptif

La prise de contraception est une décision personnelle qui permet de prévenir des grossesses non désirées et des ITSS. Lorsqu'un partenaire vous empêche d'utiliser la contraception comme vous le souhaitez, il s'agit de *sabotage contraceptif*.

Dans cet exemple, le partenaire de Margot ne souhaite pas nécessairement qu'elle devienne enceinte. Il souhaite plutôt contrôler la relation sexuelle de sorte à accroître son plaisir sexuel, sans égard à la santé sexuelle et reproductive de Margot.

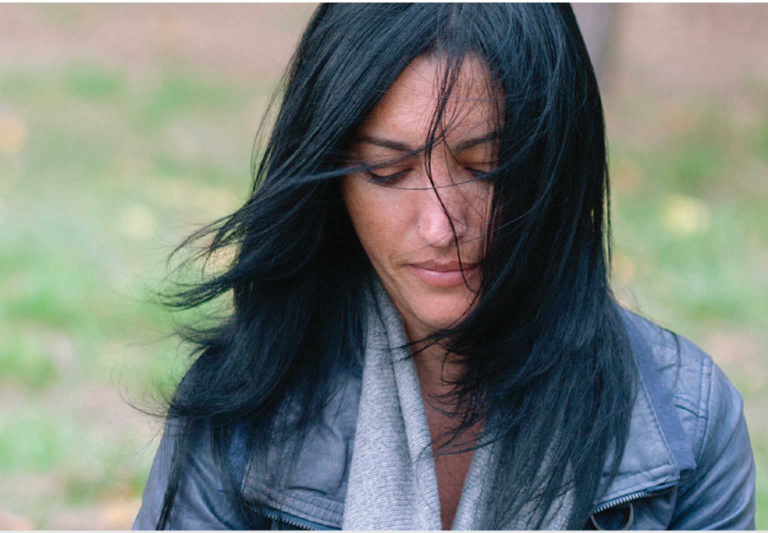

Margot a un nouveau partenaire depuis quelques mois. Ils ne forment pas un couple, mais ont des relations sexuelles ensemble à l'occasion. Margot ne veut pas devenir enceinte, alors elle utilise le condom lors des relations sexuelles. Elle a toutefois l'impression qu'à chaque relation sexuelle avec son partenaire, elle doit le convaincre d'utiliser un condom. De façon générale, il accepte de l'utiliser, un peu à contrecœur. Il lui dit souvent qu'il trouve la sensation moins agréable. Lors de leur dernière relation sexuelle, Margot a réalisé qu'il avait retiré le condom sans l'aviser. Elle est maintenant inquiète d'avoir une ITSS ou une grossesse non désirée.

Si vous avez déjà vécu ce type de situation, n'hésitez pas à en parler à un·e professionnel·le de la santé ou un·e intervenant·e (des ressources sont disponibles à la fin du livret). La prise de contraception et le port du condom sont des décisions personnelles qui permettent de prévenir des grossesses non désirées et des ITSS. Cette personne pourra vous informer des méthodes de contraception qui sont peu détectables par votre partenaire (p. ex. : le stérilet). Elle pourra aussi vous mettre en contact avec un·e intervenant·e qui connaît bien le type de situation que vous vivez.

Forcer l’autre à avoir un enfant contre son gré, est-ce un signe d’amour ?

La décision d’avoir ou non un enfant peut parfois faire l’objet de discussions difficiles avec votre partenaire. Toutefois, il y a une distinction entre être en désaccord et mettre de la pression sur l’autre personne pour l’obliger à agir comme on le souhaite.

Pour vous aider dans votre réflexion, vous pouvez vous demander si ces phrases reflètent ce qui se passe avec votre partenaire :

- Il arrive que votre partenaire vous mette de la pression pour que vous deveniez enceinte, alors que vous ne le voulez pas.

- Votre partenaire vous a déjà blessée ou vous a menacée parce que vous ne vouliez pas devenir enceinte.

- Votre partenaire vous a déjà dit qu’il allait vous blesser si vous ne faisiez pas ce qu’il voulait avec la grossesse (p. ex. : vous obliger à poursuivre la grossesse ou vous obliger à avorter).

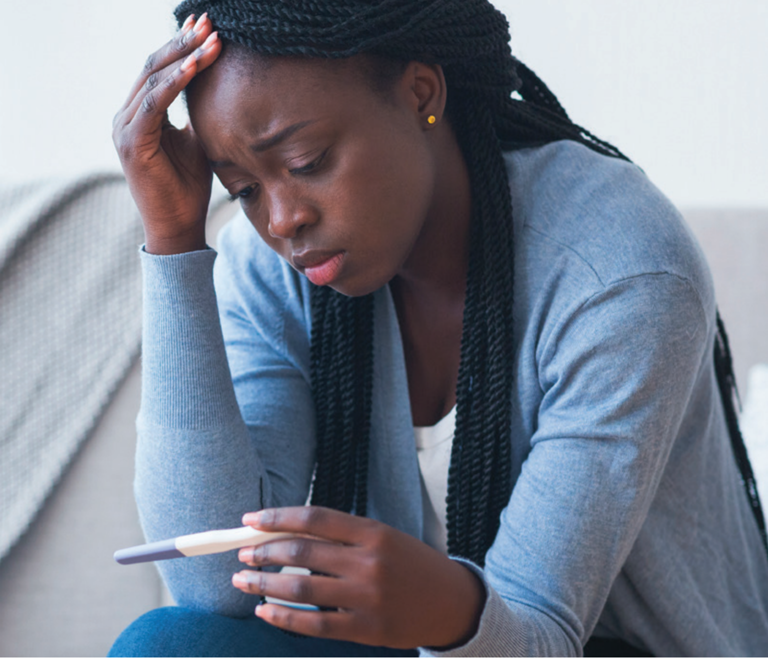

Si vous avez répondu « oui » à l'un ou plusieurs de ces énoncés, votre relation présente des signes de *coercition reproductive*, une forme de violence entre partenaires intimes qui interfère avec la contraception et la planification des naissances. Forcer l'autre à avoir un enfant contre son gré n'est pas un signe d'amour ou d'engagement dans la relation.

Votre partenaire ne peut pas vous obliger à devenir enceinte contre votre gré, ou à poursuivre une grossesse que vous ne voudriez pas mener à terme.

Exemple de situation qui présente de la pression liée à la grossesse et des manifestations de violence psychologique

Les relations intimes et amoureuses évoluent avec le temps. Parfois, de nouvelles situations ou de nouvelles discussions entraînent des conflits dans le couple. Il est possible aussi que ces conflits soient de plus en plus violents. Cet exemple révèle des situations de *violence psychologique* et de *pression liée à la grossesse* perpétrées par Michel à l'égard d'Anna.

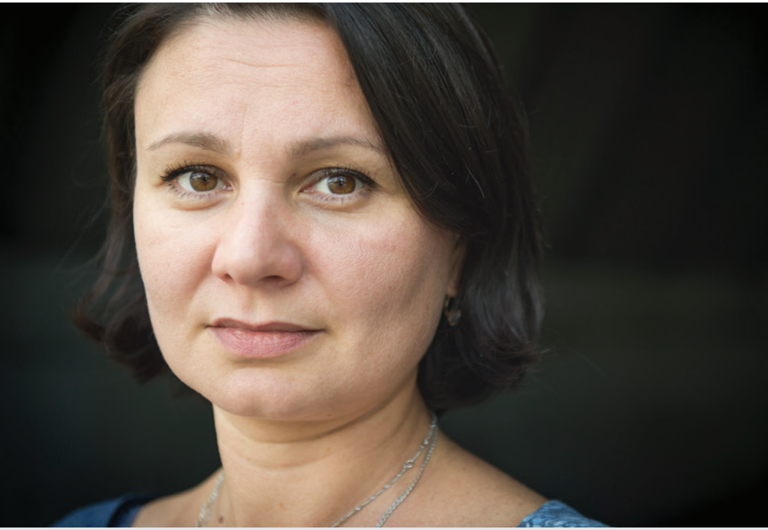

Forcer l'autre à avoir un enfant contre son gré, est-ce un signe d'amour ?

Anna est mère de deux enfants issus d'une union précédente. Elle est en couple depuis trois ans avec Michel. Depuis plusieurs mois, Michel dit à Anna qu'il souhaite avoir un enfant avec elle. Toutefois, Anna ne veut plus d'enfant et lui a dit. Il persiste tout de même et devient de plus en plus insistant. Lorsque Anna rencontre une intervenante dans un organisme communautaire, elle lui confie que Michel lui dit souvent: « Si tu m'aimais vraiment, tu voudrais un enfant avec moi. J'en déduis donc que tu ne m'aimes pas autant que moi je t'aime ». Anna se sent coincée.

Si vous avez déjà vécu l'une ou plusieurs de ces situations avec votre partenaire, parlez-en à un·e professionnel·le de la santé ou un·e intervenant·e (des ressources sont disponibles à la fin du livret). Cette personne pourra vous écouter, vous soutenir et — si vous le souhaitez — vous orienter vers les options qui s'offrent à vous.

Pour se renseigner ou recevoir de l'aide

Si vous avez besoin de parler, de poser des questions ou si vous souhaitez avoir accès à des services d'écoute ou de santé (dépistage d'ITSS, test de grossesse, contraception d'urgence et contraception, etc.), ces ressources sont disponibles. Les services sont gratuits et confidentiels.

SOS VIOLENCE CONJUGALE
Accessible partout au Québec | 1 800 363-9010

Écoute téléphonique : pour du soutien et de l'information, disponible en anglais et en français, 24 h sur 24 et 7 jours sur 7.

GROSSESSE-SECOURS
Accessible partout au Québec, locaux à Montréal
514 271-0554 | Sans frais : 1 877 271-0555

Écoute téléphonique : toute personne préoccupée par une grossesse peut téléphoner du lundi au vendredi de 9 h à 17 h.

Clavardage en ligne : service de soutien et d'information en ligne pour toute personne préoccupée par une grossesse. Offert du lundi au vendredi de 9 h à 16 h 30.

Test de grossesse et rencontre avec une intervenante : pour les personnes de tout âge qui souhaitent passer un test de grossesse ou rencontrer une intervenante sur place. Il est préférable de fixer un rendez-vous.

SOS GROSSESSE

Accessible partout au Québec, locaux dans la Ville de Québec | 418 682-6222 | Sans frais : 1 877 662-9666

Écoute téléphonique : s'adresse à toute personne s'inquiétant d'être enceinte, préoccupée par une grossesse ou ayant vécu une fausse couche. Offert tous les jours de l'année de 9 h à 21 h.

Clavardage en ligne : service de soutien et d'information à toute personne préoccupée par une grossesse.

Test de grossesse et rencontre avec une intervenante : pour les personnes de tout âge qui souhaitent passer un test de grossesse ou rencontrer une intervenante sur place. Il est préférable de fixer un rendez-vous.

VIOLENCE INFO

Capitale Nationale | 418 667-8770

Écoute téléphonique : pour toute personne désirant se renseigner sur la violence conjugale ou partager des difficultés et des préoccupations en lien avec cette problématique de manière confidentielle. Offert du lundi au vendredi de 8h30 à 12h et de 13h à 16h30.

Intervention individuelle : rencontres avec une intervenante pour discuter de son vécu de violence conjugale, mieux comprendre la problématique, améliorer l'estime de soi, et favoriser des relations saines et égalitaires.

Intervention de groupe : espace d'échange et de prise de parole visant à partager les problématiques vécues et à reprendre du pouvoir sur sa vie.

TEL-JEUNES

Accessible partout au Québec | 1 800 263-2266

Tel-Jeunes permet à tous les jeunes de 20 ans et moins du Québec de parler avec un·e intervenant·e de ce qui les préoccupent.

Écoute téléphonique : 1 800 263-2266. Disponible tous les jours, 24 h sur 24 et 7 jours sur 7.

Texto : 514 600-1002. Disponible tous les jours, entre 8 h et 22 h 30.

Clavardage en ligne : disponible tous les jours, entre 8 h et 22 h 30.

JEUNESSE, J'ÉCOUTE

Accessible partout au Canada | 1 800 668-6868

Jeunesse, J'écoute est accessible 24/7 et offre des services de soutien en français et en anglais.

Écoute téléphonique : 1 800 668-6868. Disponible tous les jours, 24 h sur 24 et 7 jours sur 7.

Texto : envoie le mot PARLER au 686868. Disponible tous les jours, 24 h sur 24 et 7 jours sur 7.

REGROUPEMENT QUÉBÉCOIS DES CENTRES D'AIDE ET DE LUTTE CONTRE LES AGRESSIONS À CARACTÈRE SEXUEL (CALACS)

Accessible partout au Québec | 1 888 933-9007

Référence téléphonique : permet de trouver le CALACS le plus près du lieu de résidence. Il est aussi possible d'avoir des rencontres individuelles avec une intervenante qualifiée, de participer à des groupes de soutien, de recevoir de l'information et de l'accompagnement en lien avec le

système judiciaire ainsi que de l'information et du soutien pour remplir le formulaire de l'Indemnisation des victimes d'actes criminels (IVAC).

FÉDÉRATION DU QUÉBEC POUR LE PLANNING DES NAISSANCES (FQPN) | En ligne

Référence en ligne : pour en apprendre davantage sur la contraception, les grossesses non planifiées, la fertilité et l'infertilité, la justice reproductive et plus encore, consultez le site web de la FQPN au fqpn.qc.ca.

Vous trouverez aussi sur le site un répertoire de ressources en santé sexuelle et reproductive sur l'ensemble du territoire québécois en cliquant sur l'onglet « trouver de l'aide ». Vous pourrez entre autre trouver une liste des ressources offrant des services d'avortement partout sur le territoire québécois.

INFO-SANTÉ ET INFO-SOCIAL

Accessible partout au Québec | 811

Référence téléphonique : Le volet Info-Santé permet de joindre rapidement une infirmière en cas de problème de santé non urgent. Vous pourrez aussi avoir de l'information sur les cliniques offrant des services d'avortement partout sur le territoire québécois. Le volet Info-Social permet de joindre rapidement un·e intervenant·e en cas de problème psychosocial. Au besoin, une ressource appropriée sera proposée dans le réseau de la santé et des services sociaux ou une ressource communautaire. Le service est offert 24 heures par jour, 365 jours par année.

Cette brochure est un complément à l'outil Coercition reproductive et violence entre partenaires intimes. Un guide d'intervention destiné aux professionnel·le·s de la santé et aux intervenant·e·s.

© Sylvie Lévesque, UQAM

Dépôt légal
Bibliothèque et Archives nationales du Québec, 2021
ISBN : 978-2-9818518-2-6

UQÀM Québec
